# Non‐Linear Increases of Soil Carbon Stocks and Turnover Along Alpine Greening Gradients on Different Bedrocks

**DOI:** 10.1111/gcb.71158

**Published:** 2026-10-22

**Authors:** Annegret Udke, Kyra Marty, Cédric Bührer, Luisa Minich, Michael Zehnder, Marco Griepentog, Sebastian Doetterl, Negar Haghipour, Timothy Eglinton, Christian Rixen, Markus Egli, Frank Hagedorn

**Affiliations:** ^1^ Swiss Federal Institute for Forest, Snow and Landscape Research WSL Birmensdorf Switzerland; ^2^ Department of Geography University of Zurich Zurich Switzerland; ^3^ Department of Environmental Systems Sciences ETH Zurich Zurich Switzerland; ^4^ Department of Earth and Planetary Sciences ETH Zurich Zurich Switzerland; ^5^ WSL Institute for Snow and Avalanche Research SLF Davos Switzerland; ^6^ Climate Change, Extremes and Natural Hazards in Alpine Regions Research Centre (CERC) Davos Switzerland

**Keywords:** alpine greening, bedrock/parent material, C input, C saturation, elevation gradient, radiocarbon, SOC dynamics, SOC stocks, SOC turnover

## Abstract

Alpine ecosystems are experiencing warming, leading to alpine greening—an upward migration and enhanced productivity of vegetation. Although soils represent the largest carbon (C) stocks in these ecosystems, impacts of greening on soil organic carbon (SOC) dynamics remain unknown, especially in the high alpine zone. We measured SOC stocks, quality and radiocarbon (^14^C) based turnover rates along elevation gradients from (sub)alpine forests to periglacial zones on three bedrock types, representing trajectories of changes in climate and vegetation and weathering regime. Data were linked to SOC stabilizing pedogenic oxides (PO) to assess the potential of soils to sequester C upon alpine greening. Results show pronounced decreases in SOC stocks (from 10 to 1 kg C m^−2^) and increases in ^14^C‐based turnover times (from decades to millennia) with increasing elevation. This pattern followed a non‐linear sigmoidal function with a threshold at the vegetation line where grassland transitions to the nival zone. High alpine soils showed low SOC:PO ratios, indicating low C saturation and a high capacity to stabilize SOC on reactive minerals. However, negligible ^14^C‐derived carbon inputs suggest that low vegetation productivity constrains SOC sequestration in these soils. In contrast, towards lower elevation with a warmer climate, grassland soils receive higher C inputs, but appear more C saturated, leading to greater SOC turnover but little SOC stock changes. Bedrock influenced SOC dynamics with approximately 50% smaller SOC stocks and faster turnover rates in dolomitic than in siliceous soils, due to slower weathering and lower SOC stabilization. Using elevation as a space‐for‐time substitution, we infer that SOC sequestration under alpine greening will be confined to areas around the vegetation line, with parent material influencing the magnitude of C uptake. Future projections of SOC dynamics should account for vegetation shifts and bedrock‐driven stabilization to constrain the role of alpine soils in land carbon‐climate feedbacks.

## Graphical abstract

Alpine warming and greening have largely unknown consequences for soil organic carbon (SOC) dynamics and the mechanisms governing sequestration potential. Along elevation gradients from subalpine forests to the periglacial zone on three bedrock types, we show that SOC stocks and radiocarbon (^14^C)‐derived turnover times follow a non‐linear sigmoidal relationship with elevation, with a distinct threshold at the vegetation line. High alpine nival soils receive negligible carbon inputs, reflected in millennia‐long ^14^C‐derived turnover times and low SOC saturation relative to SOC‐stabilizing pedogenic oxides (PO). Downslope grassland soils receive higher carbon inputs and are therefore more C‐saturated, driving faster turnover with little net SOC gain. We infer that SOC sequestration potential under alpine greening will be confined to areas around the vegetation line, with bedrock type modulating the magnitude of carbon uptake.

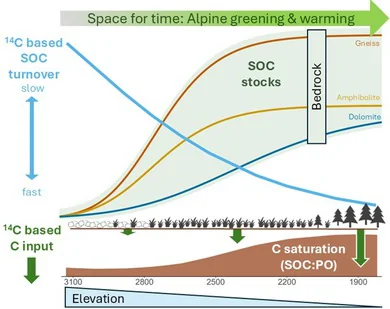

## 1 Introduction

Alpine ecosystems are currently experiencing pronounced environmental changes driven by the strong warming at high elevation (Nogués‐Bravo et al. 2007; Rangwala et al. 2013; Pepin et al. 2015, 2022) and land abandonment leading to changing land cover (Gehrig‐Fasel et al. 2007; Mietkiewicz et al. 2017). As a consequence, vegetation productivity is increasing, and plant species and communities are migrating to higher elevations (Leonelli et al. 2011; Steinbauer et al. 2018). While alpine greening is well documented (Rumpf et al. 2022; Choler et al. 2024), substantially less is known about how these climatic and vegetation changes are affecting soils despite their crucial role in ecosystem functioning, including carbon (C) storage, nutrient provisioning, slope stabilization, and harboring biodiversity (Hagedorn et al. 2019).

Soil organic matter (SOM) is of fundamental importance to all these soil functions. SOM has a highly heterogeneous nature, consisting of a multitude of components which turn over on daily to millennial timescales, involving multiple processes that decompose and transform plant‐derived C detritus into stable soil organic carbon (SOC) (Lehmann and Kleber 2015; Moreno‐Duborgel et al. 2025). Pathways of SOC cycling, such as microbial decomposition as well as the stabilization and preservation on mineral surfaces (Lavallee et al. 2020), are strongly controlled by climatic conditions (e.g., temperature and precipitation; Garcia‐Pausas et al. 2007; Yang et al. 2008), the amount and quality of the plant litter input (Djukic et al. 2010; Enri et al. 2021), but also soil geochemical properties, such as the reactivity of mineral surfaces (Maier et al. 2025; Doetterl et al. 2015).

Ongoing changes in climate and land‐use are impacting these factors and their interactions (Hagedorn et al. 2019; Bright et al. 2025). In the Alps, elevational shifts of the vegetation zones due to warming—encompassing subalpine forests, alpine tundra, and grasslands to nearly vegetation‐free periglacial environments (Körner 2003)—are accompanied by enhanced plant productivity (Hagedorn et al. 2019; Rumpf et al. 2022), which increases C inputs into the soil (Yan et al. 2022, Ma et al. 2022), and alters the quality of litter inputs to the soil (Solly et al. 2017; Hagedorn et al. 2025). However, the enhanced C inputs under a warmer climate may be at least partially balanced out by accelerated microbial decomposition, releasing C to the atmosphere (Streit et al. 2014; Chen et al. 2020). Indeed, studies along elevational gradients in alpine grasslands and across treelines show that changes in vegetation and climate with elevation impact SOC turnover and properties (Leifeld et al. 2009; Budge et al. 2011; Kammer et al. 2009). However, the magnitude and patterns of net effects on SOC stocks remain uncertain (Bright et al. 2025). Additionally, SOC stocks and cycling are influenced by parent material and mineralogy along with weathering regimes, controlling the stabilization and long‐term preservation of SOC through the interaction of organic matter with reactive mineral surfaces from pedogenic oxides and clay (Dümig et al. 2011; Maier et al. 2025). A recent study in alpine grasslands even suggests that parent material is even more important for SOC storage than plant inputs (Maier et al. 2025).

In alpine ecosystems above the treeline, soils typically store more than 90% of the ecosystem's carbon (Körner 2003; Hagedorn et al. 2019). Nevertheless, our quantitative understanding of SOC cycling in alpine soils remains limited (Bright et al. 2025). Research on alpine soils is confronted with difficult accessibility, a limited socio‐economic interest in these harsh environments, a high spatial variability, and stony nature of soils, making soil sampling labour‐intense. Consequently, previous studies in the European Alps predominantly focused on topsoils in the accessible lower alpine belt (i.e., elevations below 2300 m a.s.l., Leifeld et al. 2009; Budge et al. 2011; Maier et al. 2025) while estimates of full profile SOC stocks, particularly at higher elevations around the vegetation line, remain scarce. While SOC accumulation and cycling with ecosystem development has been studied along glacier forefields (Smittenberg et al. 2012; Guelland et al. 2013), almost no information exists on SOC cycling in the nival and periglacial zone above the vegetation line. Here, vegetation and microbial communities extend to substantially higher elevations and, in contrast to recently deglaciated pro‐glacial areas, soils have developed since the last ice age and not only for the last centuries. Nevertheless, topsoils in the high elevation periglacial region of mountain ecosystems are almost SOC‐free due to the absence of vegetation input (Adamczyk et al. 2021; Pascual et al. 2021; Kuhry et al. 2022; Fröjd et al. 2022; Fuchs et al. 2015). This suggests that, along the succession from the periglacial zone to grasslands, soils could sequester more SOC and act as C sinks as vegetation shifts upward under climate change driven alpine greening (Hagedorn et al. 2019). Long‐term soil C sequestration in these environments also depends on the availability of reactive mineral surfaces formed through climate‐ and bedrock‐specific weathering (Fang et al. 2019; Jia et al. 2024). Moreover, the SOC build‐up with increased vegetation inputs progressively “saturates” mineral sorption sites (Georgiou et al. 2025), thereby reducing the soil's capacity to store additional C (Stewart et al. 2007). In alpine regions, however, the magnitude and speed of SOC storage and cycling changes remain poorly understood, limiting predictions of future SOC dynamics.

Our study aimed at estimating SOC stocks, turnover, and stability in alpine soils under future warming and greening trajectories. Therefore, we applied a space‐for‐time substitution approach and sampled soils along three 1400 m elevational gradients ranging from sub‐alpine forests to the periglacial vegetation‐free zone on gneissic, amphibolitic, and dolomitic bedrock. To elucidate SOC turnover and persistence across varying timescales and to explore how rapid changes in SOC may take place, we measured radiocarbon (^14^C) in soil profiles and in respired CO_2_ along the gradients. Using modeling approaches, ^14^C content in soils can be transferred into mean system ages, reflecting the average time that a C atom spends in the soil system since entering (Sierra et al. 2017). As mean system ages of bulk soils integrate over different SOC pools (Moreno‐Duborgel et al. 2025), we used ^14^C in microbially‐respired CO_2_ (Sierra et al. 2017) to identify the turnover of the actively cycling SOC pool (Trumbore 2000, 2009; Minich et al. 2026). The ^14^C measurements were combined with quantification of SOC stocks and its biodegradability, in situ soil CO_2_ fluxes, as well as pedogenic oxides, a measure of SOC stabilizing, reactive mineral surfaces. This allows a comprehensive understanding of the current SOC cycle and inferring possible future responses of SOC to ongoing warming and greening in alpine environments. We hypothesized that SOC storage would increase from the periglacial across the nival and grassland zone to the subalpine forest, with the magnitude of this increase depending on bedrock type, which in turn drives plant productivity, weathering, and soil development. We also expected that SOC turnover would accelerate along the same trajectory—even more strongly than SOC stocks—as rising temperature affects both C in‐ and outputs and reactive mineral surfaces become increasingly saturated with SOC. The results provide novel insights into the long‐term potential and limitations of alpine soils to sequester atmospheric C in a warmer and greener future.

## 2 Methods

### 2.1 Study Sites

Three elevation gradients (1750–3140 m a.s.l.) were sampled near Davos, Switzerland (Figure S1, Table S1), where MAAT ranges from 3.7°C to −3.2°C and mean annual precipitation averages 1622 mm (Table S2; IMIS 2023, SwissMeteo 2024). Sites lie within the Silvretta nappe, characterized by Paleozoic–Mesozoic parent materials ranging from crystalline to sedimentary rock (Maggetti and Flisch 1993). We distinguished three main bedrock types: dolomitic (main dolomite), gneissic (gneiss and mica schist), and amphibolitic (metamorphic basalt) (Geo500, SwissTopo 2024). Two additional high‐elevation gneissic sites were sampled in the Bernina region (~40 km SW) to complete the gradient, where MAAT is −2.7°C and MAP is 1335 mm (SwissMeteo 2024). Four ecosystem belts were represented along the gradients: (i) old‐growth (sub)alpine forests with trees, shrubs, and ericoids; (ii) alpine grasslands with forbs and graminoids; (iii) sparsely vegetated nival zones (rock cover > 60%); and (iv) periglacial blockfields with lichens (Figures S2, S3). Forest and grassland sites are nowadays seasonally grazed by cattle and sheep. Sites were chosen in the field to be representative of the local condition. Forest sites were dominated by (slightly) podsolised Humic Cambisols, while grassland sites were characterized by Umbric Regosols, which transitioned to Ochric Regosols in the nival zone. Soils on dolomitic bedrock were primarily Calcaric Cambisols and Regosols. Study sites spanned a range of geomorphic positions from summits and interfluves to planar and convex midslopes. Freeze–thaw processes likely appear across all sites with one nival site showing evidence of active solifluction.

### 2.2 Field Sampling

Soil temperature at 6 cm depth was monitored between 2022 and 2024 using TSM loggers (TMS‐4 Standard, TOMST, Czech Republic), except in non‐vegetated, stony sites. Plant species relevés were performed at each site (Table S3), recording total and species‐level cover (Figures S2, S3). Aboveground biomass from ground covering vegetation was determined from two 20 × 20 cm vegetation samples per site, oven‐dried and weighed.

### 2.3 Soil Sampling and Processing

At each site, one soil pit was excavated down to the underlying parent material (at 25 to 120 cm depth). Due to the stony nature of the soils, soil thickness was determined from the maximum possible excavation depth at the site. Mean soil depth (Table S1) was 70 cm, deepest in forests (85 cm) and shallower in grassland, nival, and blockfield sites (65 cm). Dolomitic soils were shallower (63 cm) than gneissic (75 cm) and amphibolitic sites (71 cm). In forest soils, all sites showed organic layer horizons, ranging from 0.5 to 15 cm thickness. Samples were taken volume proportionally in pre‐defined depth intervals (0–5, 5–10, 10–20 cm, then every 20 cm) and plumb‐vertically (Prietzel and Wiesmeier 2019). Two additional field replicates were collected for 0–5 and 5–10 cm depths, including the organic layer if present. All soil material, including stones, roots and the fine earth, was excavated in the pre‐defined depth intervals. Sampled soil volumes were measured (950 to 20,000 cm^3^), the soil material weighted, homogenized and sieved to 4 mm in the field to separate stones (> 4 mm) from soil. When large stones risked the collapse of the pit, their volume was estimated from their approximate geometry and considered in SOC stocks calculations. Roots were removed by hand picking during the sieving process and subsequently used to determine belowground biomass. Aliquots of the < 4 mm fraction were stored at 4°C and about 100 g were freeze dried, sieved to 2 mm and milled for SOC analysis, while about 200 g were oven dried at 60°C for basic soil physico‐chemical analysis. Remaining soils were frozen for analysis requiring fresh samples.

### 2.4 In Situ Soil CO_2_ Fluxes

Soil respiration (SR, soil CO_2_ flux) was measured in situ at all sites during four campaigns (September 2022; July, August and September 2023), each completed within 3 days to minimize weather‐related variability. At each site, soil CO_2_ flux was recorded at three locations using a 20 cm diameter chamber for 5 min, along with air moisture and temperature (GMP70, Vaisala). Rings were installed immediately before the measurement to prevent disturbance by livestock. SR was calculated from the linear increase in CO_2_ concentration over time. For site comparisons, SR was normalized to 10°C (R10):

(1) $$R10=\mathrm{SR}*Q10^{\frac{10-T_{\text{meas}}}{10}}$$

where *Q*10 (temperature dependency of SR) was assumed to be 3 (measured at treeline in the study area, Hagedorn et al. 2010) and $T_{\text{meas}}$ is the soil temperature during the measurement.

### 2.5 Laboratory Analysis

#### 2.5.1 Basic Soil Parameters

Soil pH was measured potentiometrically at room temperature using a glass electrode (BlueLine 22 pH by Schott AG) after mixing 10 g of oven dried soil and 20 mL of 0.01 M CaCl_2_. Sand (2 mm–63 μm), silt (63–2 μm) and clay (< 2 μm) content was measured for samples in the 5–10 cm depth interval and for one subsoil sample (40–60 cm or 60–80 cm depth, depending on profiles) using the sedimentation method (Gee and Bauder 1986) following oxidation with hydrogen peroxide (30%) to remove organic matter. Total elemental concentrations were measured in 5 g of finely milled samples using X‐ray fluorescence (XRF, Spectro Xepos, Spectro Analytical Instruments, Germany).

#### 2.5.2 SOC Content, C:N Ratio and Stable Isotopes

The total SOC and nitrogen content alongside their stable isotopes were measured in freeze‐dried and milled samples. For soils with pH > 6, samples were fumigated using the stepwise acidification method according to Walthert et al. (2010), exposing milled samples in silver capsules to the vapor of 100 mL 32% HCl in a desiccator. C, N, ^13^C, and ^15^N were determined using an automated elemental analyser (Euro EA 3000, Euro Vector) coupled with a continuous flow isotope ratio mass spectrometer (IRMS, Delta V advanced, Thermo Fisher Scientific Inc.). Results are reported using the delta (δ) notation to represent ‰ relative to VPD‐Belemnite for C and atmospheric N_2_ for N, respectively.

#### 2.5.3 SOC Stocks

SOC stocks were calculated according to Poeplau et al. (2017):

(2) $$\mathrm{SO}C_{\text{stock}}=\frac{m_{2\mathrm{mm}}}{V_{\text{sample}}}*\mathrm{SOC}*d*10$$

where $\mathrm{SO}C_{\text{stock}}$ is the SOC stock in kg m^−2^, $m_{2\mathrm{mm}}$ is the dry mass of the fraction < 2 mm in g, $V_{\text{sample}}$ is the sample volume in the field in cm^3^, SOC is the total SOC content in %, d refers to the thickness of the depth interval in cm and 10 is a conversion factor. Note that the $\frac{m_{2\mathrm{mm}}}{V_{\text{sample}}}$ term refers to the fine earth density, already excluding stones.

#### 2.5.4 Radiocarbon Content and Mean System Ages

Bulk soil ^14^C was determined in 2 mm‐sieved, milled samples acidified with 37% HCl (72 h, 60°C) and neutralized with NaOH (48 h, 60°C). Analyses were performed at ETH Zurich using a MICADAS accelerator mass spectrometer with a gas ion source (Synal et al. 2007; Wacker, Bonani, et al. 2010; Wacker, Němec, and Bourquin 2010; Ruff et al. 2010) coupled with an Elemental Analyser (EA vario MICRO cube, Elementar). Measurements are reported in ∆^14^C notation (Stuiver and Polach 1977), and measurement uncertainty was 6‰–8‰.

SOC turnover times in bulk soils were estimated with a one‐pool radiocarbon model (OnepModel14, SoilR, Sierra et al. 2014) using SOC stocks, ^14^C content and the atmospheric ^14^C timeseries from Jungfraujoch (Hua et al. 2022; Emmenegger et al. 2023). The atmospheric ^14^C record was adjusted for carbon transfer delays from vegetation to soil (Leifeld et al. 2015; Solly et al. 2013; Table S5).

The SoilR model uses an inverse parameter estimation approach where SOC in‐ and outputs are represented by linear differential equations (Sierra et al. 2014):

(3) $$\frac{\mathrm{dC}\left(t\right)}{\mathrm{dt}}=I+A*C\left(t\right)$$

where $C\left(t\right)$ is the SOC stock at time t, I is the *C* input and A is a vector for decomposition rates. For simplicity, we assume a steady‐state system where I and A are time‐invariant. Thus, the decomposition rate simplifies to A=1/τ where τ is the turnover time.

Corresponding ^14^C dynamics were expressed as:

(4) $$\frac{d^{14}C\left(t\right)}{\mathrm{dt}}=I_{14C}+A{*}^{14}C\left(t\right)-\lambda^{14}C\left(t\right)$$

where λ is the ^14^C decay constant of 1/8267 year^−1^. The best‐fit τ was chosen based on consistency with observed SOC stocks and adjacent depth layers.

Under steady‐state assumptions and within the one‐pool framework, the inferred turnover rates correspond to the mean system age, describing the average time that carbon currently stored in soil has remained in the system since the time of entry (Sierra et al. 2017). This approach is a strong simplification of SOC dynamics, as the SOC pool is treated as a homogenous pool while the ^14^C signal integrates over different pools of varying size and turnover (Moreno‐Duborgel et al. 2025). Additionally, the steady‐state assumption does not account for disequilibrium between C input and output rates, such as by a time lag between warming and upward migration of plants (Alexander et al. 2018; Liu et al. 2026). Consequently, mean system ages should be interpreted as comparative indicators of SOC turnover rather than exact C residence times.

To constrain C transit times, ^14^C of microbially respired CO_2_ was measured in gas samples retrieved from incubated (20°C, 9–33 days, combusted 2 L glass bottles) fresh, root‐free soils (4 mm sieved) from selected grassland and elevational profiles. CO_2_ was collected with the Air Loading Facility (ALF; Gautschi 2017), graphitized (AGE3; Wacker, Němec, and Bourquin 2010), and analyzed by MICADAS at ETH Zurich (uncertainty < 2‰).

#### 2.5.5 Biodegradability From Lab Incubation

We measured mineralization of topsoil SOC (biodegradability) at all sites under controlled temperature and moisture conditions in a separate incubation experiment. Two field replicates of 4 mm sieved fresh soil samples were placed in stericups (Quick Release, Millipore, USA) and put in air‐tight boxes in climate chambers maintained at 22°C. Respired CO_2_ was trapped in 25 mL 0.05 M NaOH and titrated with 0.05 M HCl on a weekly basis for 4 weeks. After each week, samples were leached with 50–100 mL of nutrient solution evacuated with a vacuum pump at 400 hPa.

#### 2.5.6 Pedogenic Oxides

To determine the amount of pedogenic metal phases (pedogenic oxides, PO) in the bulk soil material that are relevant for SOC stabilization, we carried out extractions with pyrophosphate (PY) and oxalate (OX) (Bascomb 1968; Loeppert and Inskeep 1996). Briefly, for the PY extraction, 1 g of soil (< 2 mm) was mixed with 50 mL 0.1 M tetra‐sodium pyrophosphate decahydrate and shaken for 16 h on an overhead shaker at pH 10. For the OX extraction, 1 g of soil was mixed with 50 mL 0.2 M ammonium oxalate and shaken for 4 h at pH 3 in an opaque bottle. After shaking, extracts were filtered and stored at 4°C until measurement of Al, Fe and Mn concentrations using an ICP‐OES (5100 ICP‐OES, Agilent Technologies, USA). Concentrations of Al, Fe and Mn in the PY and OX extracts are hereafter referred to as $Al_{\mathrm{py}}$, $Al_{\mathrm{ox}}$, $Fe_{\mathrm{ox}}$ and $Mn_{\mathrm{ox}}$ respectively. We use the ratio between SOC and PO as an indicator for the soil mineral SOC saturation (Khandakar et al. 2021; Wu et al. 2025), reflecting the balance between C inputs and SOC stabilizing minerals. We interpret this ratio as a relative proxy for the soils potential to stabilize incoming C rather than an absolute measure of C saturation. Factors, such as oxide composition, surface reactivity and OM layering on minerals (Kleber et al. 2021) remain unaccounted for and we therefore acknowledge the methodological and conceptual uncertainties inherent in current C saturation frameworks (Georgiou et al. 2025).

### 2.6 Data Analysis and Statistics

Data processing, visualization and statistical analyzes were conducted in R (R Core Team 2025). Unless stated differently, results were reported as mean ± standard error. The effects of elevation and bedrock on soil parameters, SOC stocks, ages and quality were tested using linear models (lm(response ~ elevation * bedrock), when only one field replica was measured) or linear mixed effect models (lme(response ~ elevation * bedrock) + (1|site), when three field replicas were measured, with site as random effect). Non‐normal residuals were square root or log transformed. *p*‐values were obtained from the lmerTest package (Kuznetsova et al. 2017), with significance set at *p* < 0.05 (Table S9). We report effect sizes using semi‐partial *R* ^2^ for linear models and partial *η* ^2^ for mixed effect models, both calculated using the effectsize package (Ben‐Shachar et al. 2020). Due to the low replication in the field, statistical models are limited in their robustness. However, the close spatial approximation of the three elevation gradients as well as the governing driver of soil physical properties on SOC dynamics allows interpreting general differences between the three elevation gradients as effects of underlying bedrock.

To test for non‐linearity along elevation gradient, we fitted generalized additive mixed effect models (gamm, R‐package gamm4, Wood and Scheipl 2025), including a smooth term for elevation, s(elevation), and the bedrock categories as fixed effects and site as random effects. For response variables with only one available measurement, we fitted simple generalized additive models (gam, R‐package mgcv, Wood 2011) considering s(elevation) and bedrock as fixed effects. In contrast to the lme models, the limited amount of sites did not allow to test for the interaction effect elevation: bedrock in a gamm as models would be under ranked. Additionally, relationships between total SOC stocks and elevation were tested separately for each bedrock type using linear models (lm) and sigmoidal models (nlsLM, minpack.lm, Elzhov et al. 2023) with the equation:

(5) $$\mathrm{SO}C_{\text{stock}}=\text{asym}/\left(1+\exp\left(\;\frac{\text{xmin}+\text{elev}}{\text{scal}}\;\right)\;\right)$$

where $\mathrm{SO}C_{\text{stock}}$ is the total SOC stock in kg m^−2^, elev is elevation in m a.s.l. and aysm, xmin and scal are model parameters. Models were fitted automatically using the Levenberg–Marquardt algorithm and evaluated visually, with adjusted *R* ^2^ (pseudo‐*R* ^2^ for the sigmoidal model) and AIC.

To test the effect of a different upslope area on SOC dynamic patterns, we calculated the contributing upslope area for each soil pit using a 2 m resolution DEM (swissALTI3D, swisstopo 2019). We applied the D‐infinity flow direction and flow accumulation tools (Hydrological toolset, Spatial Analysist, ArcGIS Pro). To test whether upslope area was related to elevation and bedrock type using the linear model from above (upslope_area ~ elevation * bedrock). Neither term nor their interaction was significant (Table S9; Figure S12). To assess whether upslope area improved model fits for SOC pools and radiocarbon content, we compared the original model (elevation * bedrock) against extended models (elevation * bedrock + upslope_area) using partial *F*‐tests, adjusted *R* ^2^, and AIC (Table S13).

## 3 Results

### 3.1 Site and Soil Characteristics

Mean summer soil temperatures (MSST) decreased with increasing elevation from 13.0°C at 2000 m a.s.l. to 7.8°C at 2800 m a.s.l. (*p_E* < 0.001). Above‐ and belowground biomass decreased nearly linearly until 2750 m a.s.l., above which total plant cover dropped from 85 to below 5% (Figure 1b,c). Graminoids and forbs dominated vegetation across most sites. Ericoid shrub species decreased with elevation, while forb cover and bare rock increased (Figure S3). Dolomitic sites showed more herbaceous plants, whereas siliceous soils had greater ericoid shrub cover and rock exposure (Figures S2, S3). Slope and aspect did not differ significantly with elevation or bedrock type (Table S9).

**FIGURE 1:**
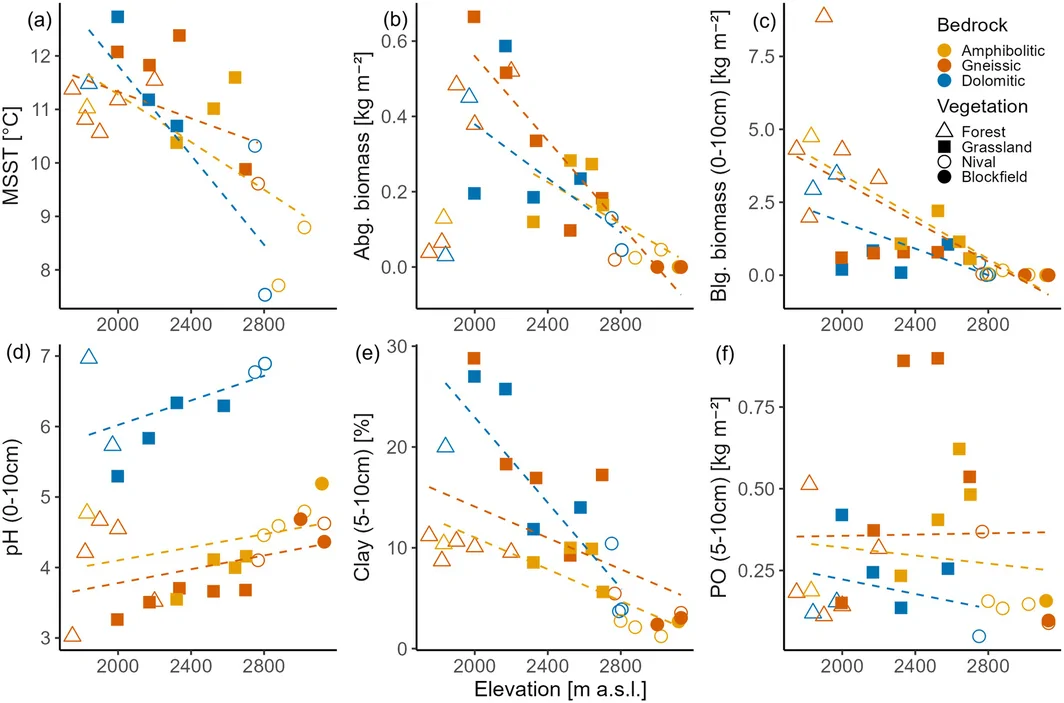
Site and surface soil characteristics, (a) mean summer soil temperature (MSST), (b) aboveground biomass, (c) belowground biomass 0–10 cm, (d) soil pH 0–10 cm, (e) clay content 5–10 cm, (f) pedogenic oxides (PO) 5–10 cm. For aboveground biomass (ground vegetation), forest sites were removed from the linear regression model shown. For *p*‐values see Table S9.

Stone contents were greater and increased more strongly with elevation in amphibolitic and dolomitic soils (*p_E:B* = 0.025), while fine earth mass was highest in gneissic grasslands. Topsoil pH (Figure 1d) increased non‐significantly with elevation but varied significantly between the bedrock types (6.5 ± 0.2 on dolomitic, 4.3 ± 0.2 on amphibolitic and 4.0 ± 0.2 on gneissic; *p_B* < 0.001). All soils were dominated by sand (77.1% ± 1.1%). Clay content (Figure 1e) was highest on dolomitic soils (14.5% ± 3.2% at 5–10 cm), followed by gneissic (11.7% ± 2.3%) and amphibolitic soils (6.3% ± 1.1%; *p_B* = 0.040), and decreased significantly with elevation (*p_E* < 0.001), e.g., from 27.0% to 4.0% along the dolomitic gradient. At 20–40 cm depth, clay content converged across bedrock types (4.1% to 4.4%). Pedogenic oxide (PO) stocks varied significantly with bedrock (*p_B* < 0.04; Figure 1f). Gneissic topsoils contained 134% and 200% more oxides than amphibolitic and dolomitic soils, respectively (Figure 1f). PO stocks peaked in grasslands at 2000 to 2500 m a.s.l. and declined at higher elevation, yet siliceous subsoils (20–40 cm, > 3000 m a.s.l.) still held 0.6 kg m^−2^, while dolomitic subsoils showed negligible PO stocks.

### 3.2 SOC Contents and Stocks

Total SOC stocks ranged from a minimum of 0.3 kg C m^−2^ in high‐elevation periglacial soils to a maximum of 20.4 kg C m^−2^ in forest soils on gneissic bedrock (Figure 2). In the latter, approximately 50% of the SOC stock existed in the organic layer. Reflecting deeper soil development and lower stone contents (Figure S11, Table S12), forest and grassland soils stored 31% of their SOC below 20 cm depth, whereas SOC stocks in the nival zone were mainly restricted to the uppermost 20 cm (Figure S4). Elevation and bedrock were both significant predictors for total SOC stocks and those in the mineral soil (*p_E* < 0.001 and *p_B* < 0.02). However, when considering only vegetated soils in grasslands and forests, we found only marginal evidence for an effect of elevation on mineral soil SOC stocks (F: 3.39; *p_E* = 0.07). Gamm models revealed the decrease in SOC stocks along the elevational gradients to be not linear (Table S12; Figure S10). On each bedrock type, mineral SOC stocks remained similar up to an elevation of approximately 2700 m a.s.l., followed by an abrupt decline towards higher elevation, when vegetation cover dropped to values below 5%. This non‐linear relationship was best represented by sigmoidal models (Figure 2, Table S4). Soils in the nival zone stored 1.9 ± 0.8 kg C m^−2^, which corresponds to about 20% of the mineral soil SOC stocks in grassland or forest sites. The threshold identified by the sigmoidal models coincided with the vegetation line identified in the vegetation surveys (Figure 2, Table S4). Splitting sites accordingly, total and mineral SOC stocks showed a significant interaction between vegetation line and bedrock (total SOC stocks: F: 3.691, *p_V:B* = 0.04, mineral SOC stocks: F: 4.00, *p_V:B* = 0.03, Table S14), indicating that the elevational pattern in SOC stocks is not a constant offset across bedrock types but differs in magnitude and shape. Surprisingly, vegetation‐free periglacial sites exhibited SOC stocks of about 0.8 ± 0.3 kg C m^−2^ underneath a stone cover of up to 1 m thickness (Figure S7). Here, we found fine textured substrate with SOC content of 0.1% to 1% which we sampled down to 80 cm depth.

**FIGURE 2:**
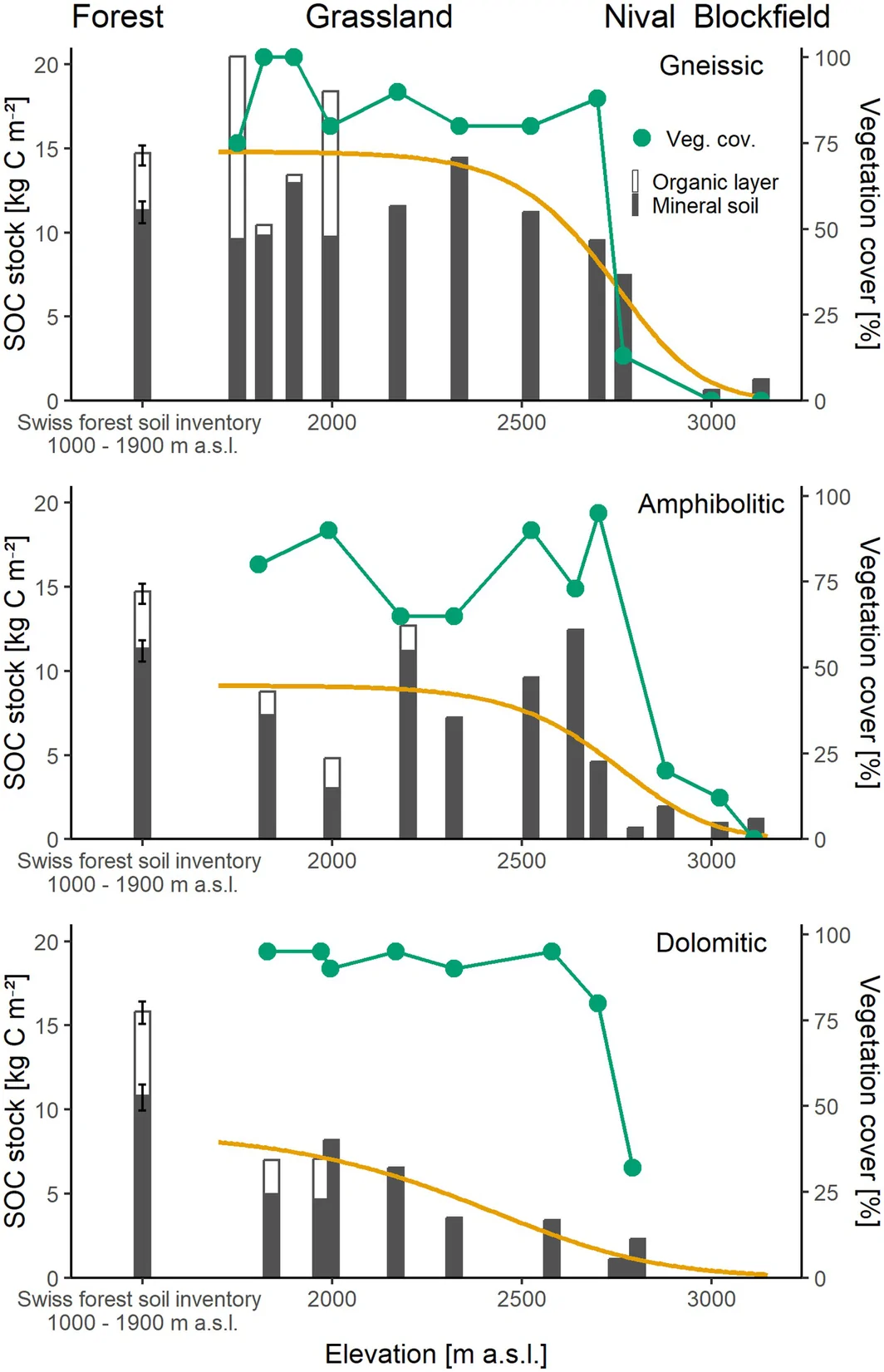
SOC stocks and ground vegetation cover along alpine elevation gradients for the three different bedrock types. The orange line represents a sigmoidal fit of total SOC stocks vs. elevation (model details Table S4). Statistical results from mixed effect models are reported in Table S9. For comparison, Swiss forest soil data for 1000–1900 m a.s.l. (Guidi et al. 2024) are shown for siliceous and calcareous bedrock types, however, do not distinguish more specific bedrock types sampled in this study.

In grassland and forest soil, bedrock significantly affected SOC stocks in the mineral soil with highest stocks on gneissic soils followed by the amphibolitic and dolomitic soils with means of 12.7 ± 1.4, 8.5 ± 1.3, and 6.0 ± 0.8 kg C m^−2^, respectively. Bedrock and elevation also affected the depth distribution of SOC stocks (*p_E* < 0.001; *p_B* < 0.001; Figure S4; Table S10). In grasslands, dolomitic soils stored on average 89% of total SOC stocks in the top 20 cm, while the gneissic and amphibolitic soils contained only 71% and 57% in the topsoil.

### 3.3 Radiocarbon Contents

Positive Δ^14^C values of bulk SOC indicated the presence of modern carbon (post‐bomb, younger than 1950; Figure S5) in the topsoil and organic layers of forest and grassland soils up to an elevation of 2500 m a.s.l. The Δ^14^C values decreased significantly with elevation (*p_E* < 0.001) and Δ^14^C depth profiles became shallower towards higher elevation. While Δ^14^C appeared to be higher for dolomitic soils, there was no significant effect of bedrock on Δ^14^C at a given soil depth (*p_B* > 0.05). However, as dolomitic soils were shallower, lowest Δ^14^C values were reached in deeper siliceous soils. Depth profiles of ∆^14^C (Figure S5) and δ^13^C (not shown) exhibit continuous vertical changes, except for one nival site.

Mean ^14^C‐based system ages, estimated with a steady‐state one‐pool model (Figure 3), revealed lowest mean system ages in the organic layer of forests (dominated by Oe layers), with an average of 13 ± 0.7 years. At 0–5 cm depth of the mineral soil, mean system ages increased slightly with elevation from the forest soils (119 ± 97 years) to grassland soils (169 ± 26 years) but increased drastically at the vegetation line, where system ages reached 2601 ± 1240 years for nival sites and 3664 ± 860 years for periglacial vegetation‐free sites. Mean system ages increased strongly with soil depth. At 60–80 cm depth, system ages reached 4274 ± 1107 years for grassland sites and 28,421 ± 12,260 years for nival sites. These old system ages were not biased by the contribution of petrogenic C, such as graphite. In selected samples on siliceous bedrock, C contents in stones were 0.04% (Table S8), which affected system ages on average only by 7% ± 2%, which appears negligible.

**FIGURE 3:**
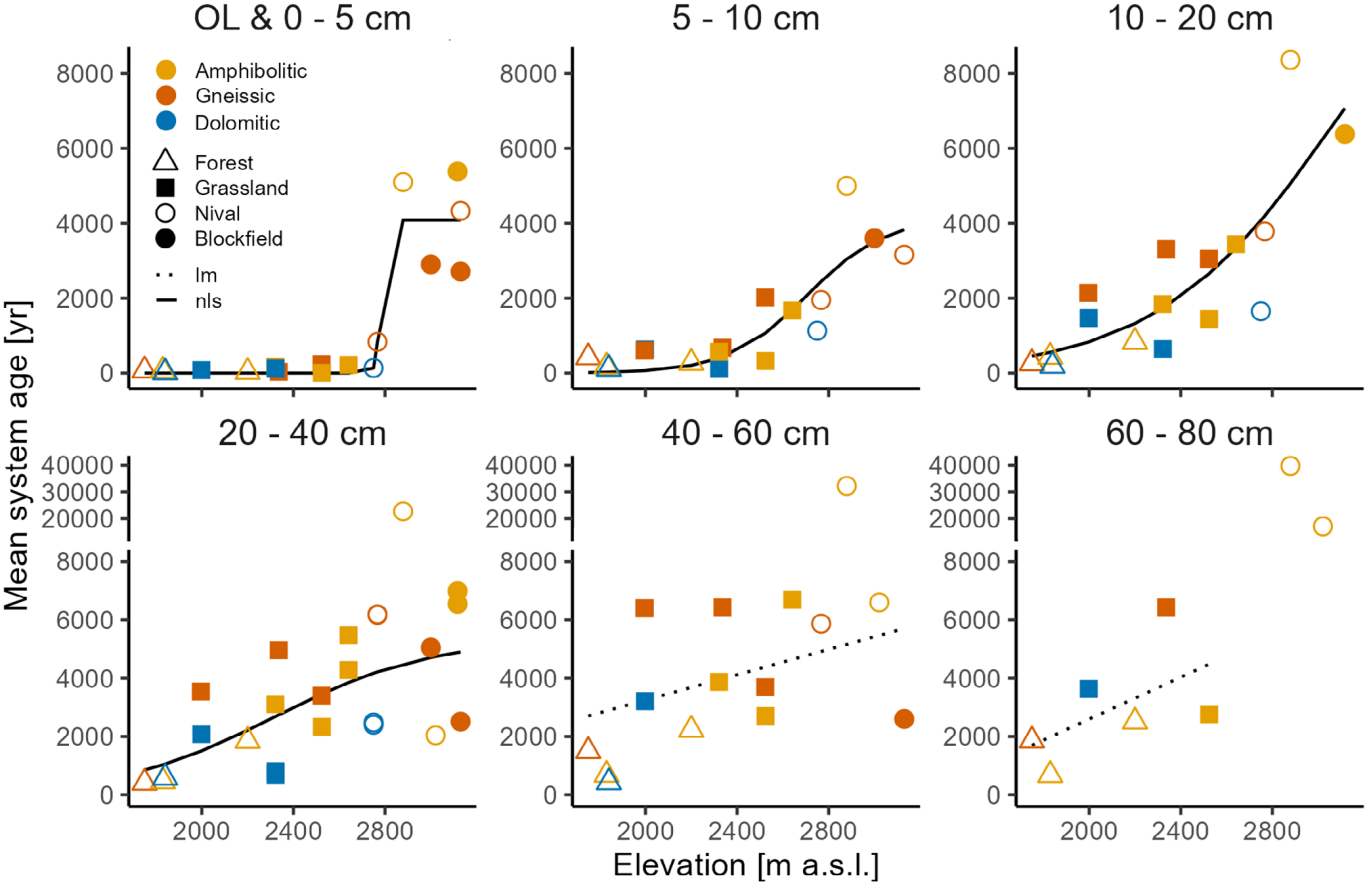
Mean SOC system ages from bulk ^14^C data across soil depth, elevation gradients and bedrock types. Solid lines show sigmoidal fits (nls), dotted lines show linear regressions (lm). Amphibolitic outliers (> 20 cm, nival) were excluded from the regression; *y*‐axis differs among panels.

The turnover model does not only calculate system ages of SOC but when linked to SOC stocks also allowed to gain insight into net C input rates into the soil. Modeled C inputs decreased with soil depth and elevation (Figure 4; Table S11). More than half (52%) of the full profile C input occurred in the organic layer and at 0–5 cm depth, while another 38% occurred at 5–40 cm. Below 40 cm, C input amounted to < 4%. There was a significant decrease of full profile C input with elevation (*p_E* = 0.008), while bedrock had no significant effect (*p_B* = 0.258). However, C inputs were shallower for dolomitic soils. Full profile C inputs decreased from forests (189 ± 25 g C m^−2^ year^−1^) to grasslands (39 ± 15 g C m^−2^ year^−1^) to nival sites (1.4 ± 0.8 g C m^−2^ year^−1^). For vegetation‐free sites, modeled C input was 0.2 ± 0.1 g C m^−2^ year^−1^. Differences between forest and grassland sites mainly arose by the high C input into organic layers in forest soils. The amphibolitic site at lowest elevation showed an extremely high C input resulting from a combination of a very high SOC stock and ^14^C value in the organic layer. The sharp decline in C input at the vegetation line was most pronounced at 0–5 cm depth, decreased within the surface soil and vanished for the subsoil > 20 cm.

**FIGURE 4:**
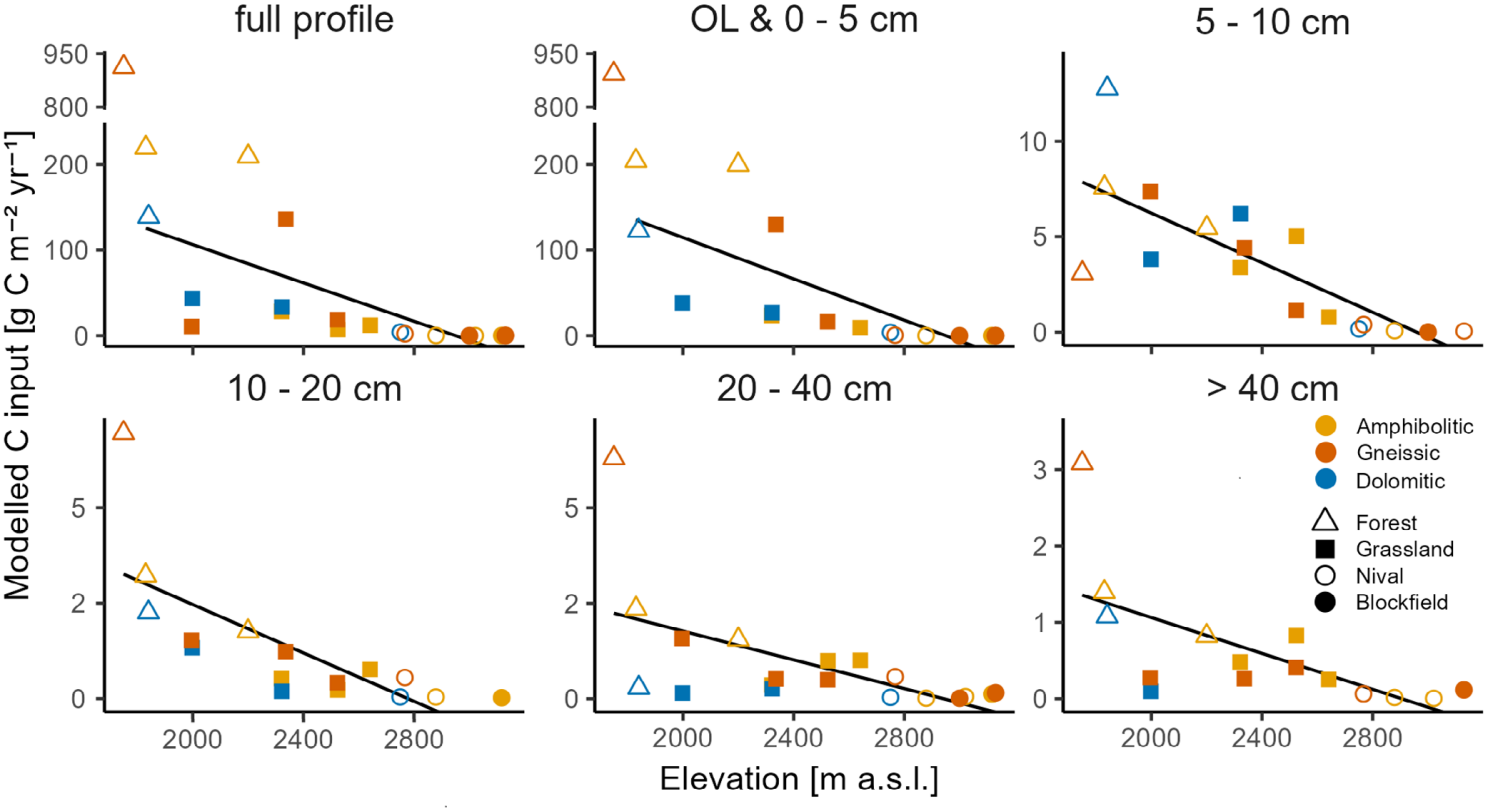
^14^C based modeled C input rates for the full soil profile and different soil depths. OL, organic layer. Black lines show linear regressions. Note varying y‐scales.

Laboratory incubations revealed consistently younger ^14^C contents in microbially respired CO_2_ compared to bulk SOC, except for the 5–10 cm sample at the dolomitic site (Figure S6). Here, alongside the low ∆^14^CO_2_ value, δ^13^CO_2_ was higher compared to the upper and lower depth layer (Table S6), possibly resulting from a higher contribution of carbonate‐derived CO_2_. All topsoil samples respired modern carbon, while pre‐modern C was respired in the subsoil, ranging from −74‰ to −138‰ in the deepest layers (40–90 cm). The differences between ^14^C signatures of bulk and respired C increased towards higher elevation with the greatest difference in the nival site (Figure S6). Additionally, differences were lowest in the dolomitic grassland soil profile compared to siliceous grasslands at similar elevation, while amphibolitic and gneissic bedrock showed similar bulk soil to CO_2_ differences (Figure S6).

### 3.4 Soil CO_2_ Fluxes and SOC Stability

Soil CO_2_ fluxes ranged between 269 mg CO_2_–C m^−2^ h^−1^ at forest sites in mid‐September and 5.3 mg CO_2_–C m^−2^ h^−1^ at high elevation periglacial sites in August 2023 (Table S7). They decreased significantly with elevation (*p_E* < 0.001), but despite about 50% lower CO_2_ fluxes along the amphibolitic transect, bedrock was not significant (*p_B* = 0.755), probably because of the high inter‐site variability for gneissic and dolomitic bedrock. Annual soil CO_2_ fluxes declined linearly with elevation and showed no effect of bedrock (Table S7). Normalizing soil CO_2_ fluxes to 10°C by calculating R10 values exhibited no significant effects of elevation or bedrock. However, relating the R10 value to SOC stocks revealed higher relative CO_2_ fluxes at similar SOC stocks for the dolomitic gradient compared to the two silicious gradients (*p_B* = 0.004; Figure 5a).

**FIGURE 5:**
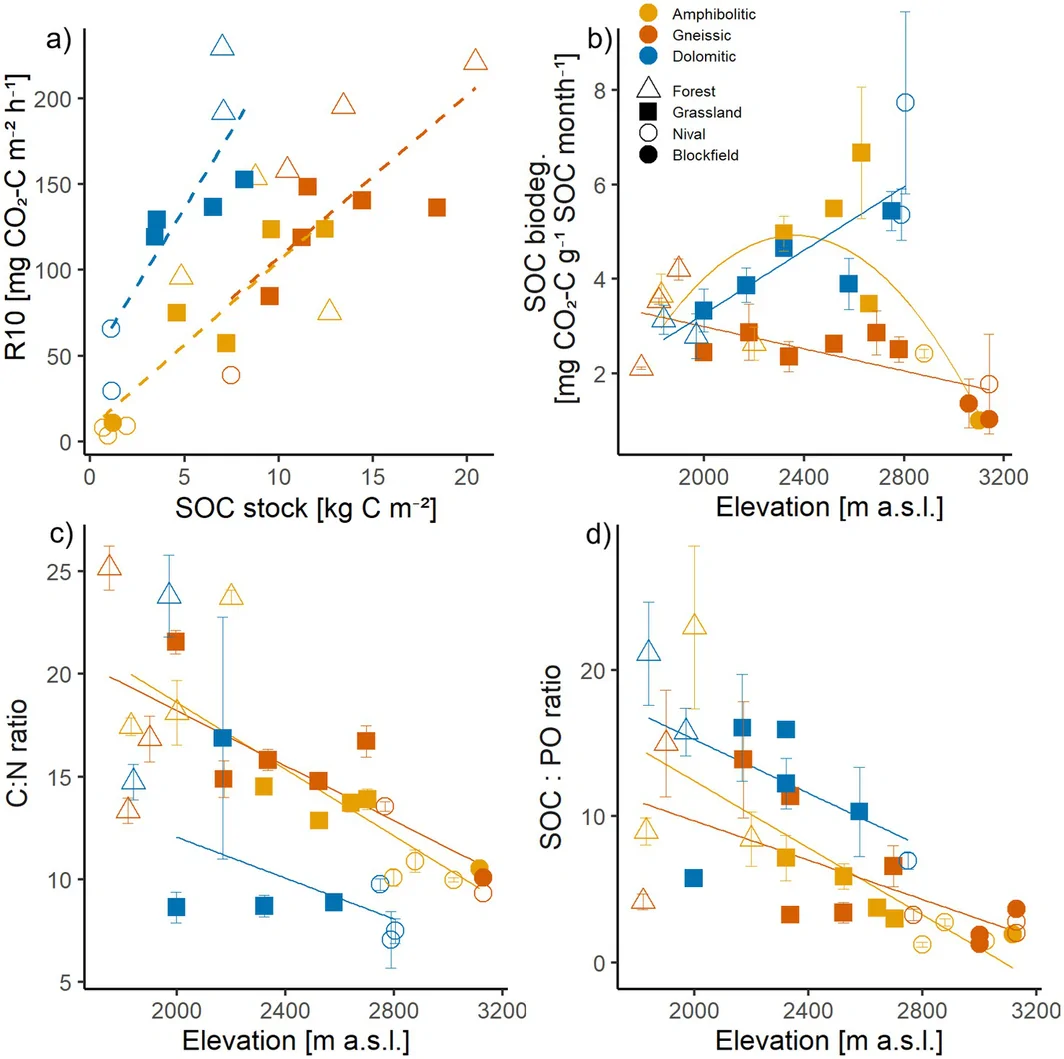
SOC quality along elevation gradients. (a) Relationship between R10 and SOC stocks, (b) SOC biodegradability (biodeg., 0–10 cm depth), (c) C:N mass ratio, 0–10 cm depth, and (d) SOC:PO mass ratio for different bedrock types. See Table S9 for *p*‐values.

SOC biodegradability, examined by relating SOC mineralization rates at 22°C to SOC contents, showed distinct elevation patterns for the different bedrock types (Figure 5b). While SOC biodegradability per g C was comparable for the forest sites (around 3.1 mg CO_2_–C g^−1^ SOC month^−1^), SOC biodegradability increased with elevation in the dolomitic soils but decreased with elevation in the soils of the two silicious bedrocks (*p_B* = 0.014; *p_E:B* = 0.004). Notably, SOC biodegradability in grasslands was higher in the amphibolitic soils than in the gneissic soils.

Along the three gradients, there was a significant decrease in C:N mass ratios with elevation (*p_E* < 0.001). The elevational pattern occurred stepwise with the shift between dominant vegetation types (Figure 5c). C:N ratios at 0–10 cm decreased from forests (19.3 ± 0.9) to grasslands (14.0 ± 0.7) and to nival and vegetation‐free sites (9.9 ± 0.5). They were slightly lower in dolomitic grassland soils in comparison to the two silicious transects but not significantly so (*p_B* = 0.900).

The ratio between SOC and PO, as a relative measure for C saturation, decreased with elevation (*p_E* = 0.037) for all three bedrock types (Figure 5d). The SOC:PO mass ratio was about two times lower above the vegetation line compared to grassland and forest sites. At the same time, dolomitic soils show 2 to 3 times higher SOC:PO ratios compared to the siliceous soils.

Mean system ages and SOC:PO ratios give insight into SOC stabilization patterns (Figure 6). Here, SOC:PO is represented by either SOC:Al_py_ or SOC:(Al_ox_ + Fe_ox_ + Mn_ox_) ratios, which capture interactions with different mineral fractions. We observed a negative exponential relation between mean system age and SOC:Al_py_ and SOC:(Al_ox_ + Fe_ox_ + Mn_ox_) ratio, which shows bedrock‐dependent clustering. Siliceous soils span a wide system age gradient at lower SOC:PO ratios, whereas dolomitic soils remain younger at higher ratios for both proxies.

**FIGURE 6:**
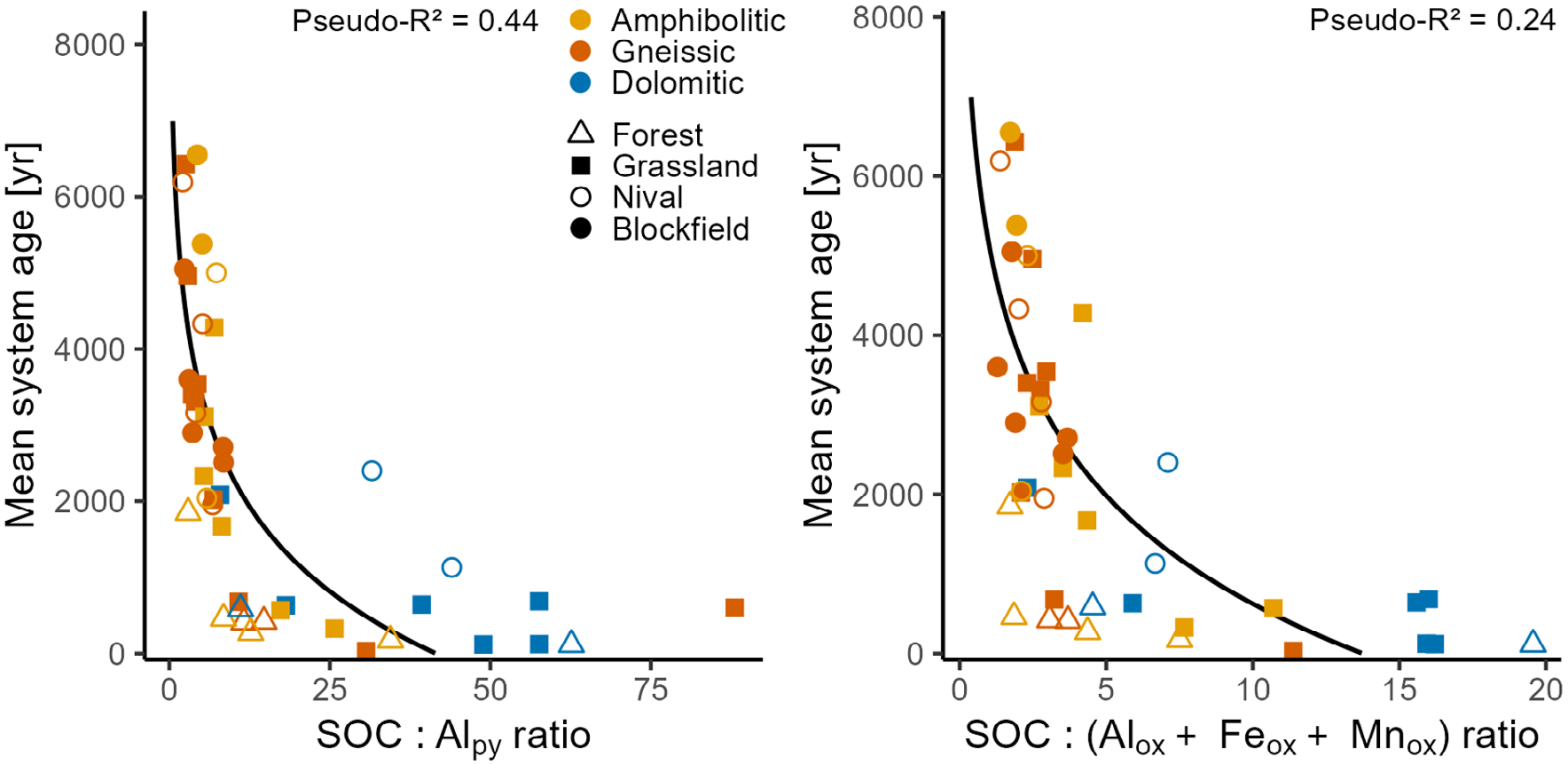
Correlations between mean system age with SOC:Al_py_ and SOC:(Al_ox_ + Fe_ox_ + Mn_ox_) mass ratios.

## 4 Discussion

Soil responses represent one key unknown in the effects of climatic warming and greening on alpine ecosystems. Here we used a space for time approach to assess impacts on SOC storage and turnover along alpine elevational gradients on different bedrock types. Our assessment reveals an increase in SOC stocks and ^14^C‐based turnover times alongside a decrease in C input rates with elevation, which shows a distinct threshold at the vegetation line. The observed patterns show that SOC dynamics are largely controlled by C input and mineral stabilization rather than by the direct effects of temperature itself. In alignment, bedrock influenced SOC dynamics with smaller SOC stocks and faster turnover in soils on dolomitic than on gneissic and amphibolitic bedrock. Results of this study provide a nuanced long‐term perspective on the C sink function of alpine soils with future warming and greening.

### 4.1 Elevational Declines in SOC Turnover

Our radiocarbon‐based assessment revealed that high alpine soils have remarkably old mean system ages signifying very slow turnover rates, and that SOC cycling changes substantially along the elevational gradients. In the nival zone (> 2800 m a.s.l.) with a plant cover of < 5% (Figure 2), system ages are older than 1000 years below 5 cm depth (Figure 3). Accordingly, ^14^C‐based C input and output rates in soils are confined to surface soils and very small, amounting to 1.4 g C m^2^ year^−1^ over entire soil profiles in these high alpine soils (Figure 4). Towards lower elevation, above‐ and belowground biomass increased strongly, accompanied by mean system ages that were several 1000 years younger and greater amounts of C that cycled through the soil (Figures 1, 4, S8). In the subalpine forest, 800 m lower in elevation, ^14^C‐based C input was approximately two magnitudes higher than in the nival zone (Figure 4). These findings are congruent with the concept that C cycling through the plant and soil system are limited by very low temperatures (MAAT of around −2.5°C in the nival zone) and are accelerated by climatic warming (Trumbore et al. 1996; Bright et al. 2025). Our results align with other ^14^C‐based case studies at lower elevations. For example, Budge et al. (2011) observed slower SOC turnover rates with increasing elevation from 2290 to 2650 m a.s.l. on an alpine grassland slope. However, the pronounced elevational pattern with very old mean system ages in high alpine soils might be reinforced by the presence of ancient SOC—a legacy of earlier warm periods in the early to mid‐Holocene, when vegetation expanded to higher elevations (Nicolussi et al. 2005; Pintaldi et al. 2021). In support, along our gradients, the high elevation vegetation‐free blockfields contained small C stocks, averaging 0.8 kg C m^−2^, with very old ^14^C ages of approx. 4000 years BP in topsoils, suggesting that parts of the SOC at high elevation sites may result from earlier warm periods. These old system ages do not reflect the incorporation of petrogenic C as recently suggested by Grant et al. (2023) as siliceous stones contained very low C contents. However, the presence of Holocene legacy carbon at high elevations could complicate the stationarity assumption underlaying the gradient study approach, which assumes that changes in SOC dynamics along elevation gradients primarily reflect differences in climate and vegetation. Additionally, time lags between warming, upward vegetation advance and soil development (Alexander et al. 2018; Hagedorn et al. 2019) introduce further uncertainty into the inference that current elevational patterns mirror future climate trajectories. Despite these limitations, the magnitude of changes in SOC cycling observed along the gradient, appears large as compared to potential legacy effects or transient disequilibria.

By measuring various C fluxes and stocks, our study documents that both rates of SOC cycling and the pathway by which C is processed in the soil are changing along the transition from unvegetated alpine terrain to subalpine forests. While soil respiration from nival soils amounted to 160 g C m^−2^ year^−1^ on average (Table S7), the ^14^C‐based model suggests that only a very small amount of 1.4 g C m^−2^ year^−1^ cycles through SOC (Figure 4). This indicates that in nival soils (> 2800 m a.s.l.), most C cycling through the soil “bypasses” the soils and leaves the soil possibly either via rhizosphere respiration or through rapid decomposition of fresh plant residues in the soil. This is further supported by low C:N ratios in nival soils (Figure 5c), indicating a high degree of microbial transformation of SOC (Springob and Kirchmann 2003). The magnitude of the ‘bypassing’ might be quantitatively biased by the disequilibrium in C inputs and outputs through a lag between upward vegetation advances (Alexander et al. 2018; Liu et al. 2026) and SOC accumulation or the presence of Holocene legacy carbon (Nicolussi et al. 2005; Pintaldi et al. 2021), which could contribute to the estimates of high system ages and slow SOC cycling rates assuming steady state conditions. Nevertheless, the over 100‐fold higher C input derived from soil respiration in comparison to ^14^C derived fluxes suggest that ‘bypassing’ likely presents the dominant process. In comparison to the nival zone, a larger fraction and amount of C inputs cycles through the SOC‐pool in grassland (on average 39 g C m^−2^ year^−1^) and forest soils (189 g C m^−2^ year^−1^), corresponding to 27‐ and 4‐times greater soil respiration than ^14^C‐based C fluxes in grassland and forest soils (Figure 4, Table S7).

This shift in the SOC cycling pathways from the nival to the vegetated zone is supported by ^14^C analyzis of microbially respired CO_2_ during lab incubations. These measurements reveal the presence of a young, fast cycling SOC pool and an old, persistent SOC pool (Figure S6). Along the elevational gradient, we found a greater difference between ^14^C in the respired flux and ^14^C in the bulk soil at the nival site compared to grassland sites, mainly due to older bulk SOC at the nival site (Figure S6), which may result from the disequilibrium discussed above. This pattern supports the increasing difference in modeled and measured C fluxes with elevation (Figure 4, Table S7). Similarly, Budge et al. (2011) found no elevational trends in turnover times in the particulate organic matter (POM) up to 2650 m a.s.l. but a slower turnover in the mineral‐associated organic matter. Additionally, our ^14^C‐based ages of microbially respired CO_2_ are younger than those reported for the POM fraction (Budge et al. 2011), supporting a rapid throughput of fresh C inputs past the soil. Overall, this suggests that C input from litter and roots is rapidly metabolized and respired independent from elevation.

### 4.2 SOC Cycling and Storage Are Shaped by Soil Carbon Stabilization Potential

In contrast to the fast‐cycling SOC pool, determined from ^14^C measurements of microbially‐respired CO_2_, the persistence of older SOC remaining in bulk soil reflects controls by mineral associations. Along the alpine elevation gradient, ^14^C‐based system ages increase as the ratio of SOC to pedogenic oxides (SOC:PO) decreases (Figures 3, 5, 6), suggesting the importance of the relative C stabilization potential in the mineral phases in regulating SOC dynamics. The pedogenic oxides analyzed here encompass organically complexed Al and non‐crystalline Al, Fe and Mn oxides (summarized as POs, Dahlgren 1994; Rasmussen et al. 2005). The role of these mineral phases in stabilizing organic matter is well documented (Porras et al. 2017; Kaiser et al. 2016), potentially involving several mechanisms including ligand exchange reactions, binding to negatively charged mineral surfaces or metal–organic complexation (Kleber et al. 2021). In the nival zone, soils are characterized by very low SOC:PO ratios (Figure 5d), indicating that these soils have a high C stabilization potential for incoming C under scenarios of alpine greening. At the same time, high mean system ages (Figure 3) suggest that the existing SOC has persisted for a long time and that the soils received very low C inputs (Figure 4). Consequently, vegetation inputs appear to be the primary factor controlling SOC stocks and cycling, as the limited organic matter supply constrains C stabilization in the soil. Conversely, lower elevation grassland and forest soils exhibit higher SOC:PO ratios (Figure 5d), indicating a lower C stabilization potential. This aligns well with previous observations of high shares of organo‐mineral associations in alpine grassland SOC (Puissant et al. 2017; Jiménez and Villar 2017; Maier et al. 2025). Younger system ages under warmer conditions at lower elevations suggest that C inputs are only partially stabilized, emphasizing, that in these soils, the limited availability of reactive mineral surfaces rather than the magnitude of C inputs primarily constrains SOC storage. This interpretation is further supported by the greater divergence in SOC:PO ratios between siliceous and dolomitic bedrock at younger system ages (Figure 6, Figure S13), pointing to bedrock‐driven mineral surface availability as a key control of SOC cycling under higher mineral C saturation. However, this pattern may be partly artefacted by complexation of Ca and Al in dolomitic soils, which can suppress Al release in pyrophosphate extractions (Ruiz et al. 2026) and thereby increase the SOC:Al_py_ ratio independently of bedrock driven mineral surface reactivity. Overall, this indicates that potentially greater C input under alpine greening may not be stabilized and therefore unlikely to increase SOC storage in alpine grassland soils.

Our results indicate that SOC cycling along alpine elevation gradients appears to be regulated less by temperature‐dependent microbial activity and more by the interplay between carbon inputs and physico‐chemical stabilization. The pronounced decline in SOC:PO ratios toward higher elevations (Figure 5d) is not primarily the result of intensified weathering or oxide formation, as POs only show minor elevational changes (Figure 1f). Instead, this reflects the limited C supply to stabilizing minerals towards the nival zone (Figure 4). Once the soil C stabilization potential is approached, SOC stocks become decoupled from turnover and fluxes. Under warmer conditions, higher carbon inputs are accompanied by increased microbial respiration and enhanced turnover, while SOC stocks remain generally unchanged (Figure 2). Mean SOC system ages therefore appear to be a consequence of the C stabilization potential, consistent with global evidence that SOC turnover is strongly constrained by C inputs and mineral saturation dynamics (Zhou et al. 2024), which are controlled by soil mineralogy (Jia et al. 2024; Zhao et al. 2019). In addition to POs, Ca can stabilize SOM via inner‐ and outer sphere complexation (Rowley et al. 2018), a mechanism not captured by the SOC:PO ratio. This limitation is particularly relevant in soils with higher pH such as the dolomitic soils, where Ca‐Ions are an abundant weathering product (Musso et al. 2022). In our study, the highest ^14^C based system ages of 3200 years in neutral to alkaline soils (Figure S9) coincided with both decreasing POs and increasing total Ca at higher pH, which provides indirect evidence for Ca‐mediated SOC stabilization. However, mean system ages are substantially older in acidic siliceous soils (8400 years) than in soils developed from carbonate‐containing parent material. We relate this to a contrast between two mineral associations as weathering lowers soil pH. Ca‐SOC bindings generally lose importance over time below pH 6.5, coinciding with the loss of soil carbonates. PO‐SOM associations, in contrast, persist and remain more resilient to acidification‐related weathering across the pH range 6–4.5 (Rowley et al. 2018). Overall, our observations highlight that SOC cycling and persistence in alpine soils are jointly regulated by carbon inputs, the soil's carbon stabilization potential, and mineral‐specific interactions.

### 4.3 Bedrock Controls SOC Stocks and Cycling

Consistent with the concept of C stabilization potential driven by mineral surface reactivity, SOC stocks and cycling depended upon bedrock types, with siliceous soils having higher contents of pedogenic oxides than dolomitic ones (Figure 1f). Accordingly, gneissic soils showed five times older system ages in surface soils (Figure 3) and twice the SOC stocks of dolomitic soils (Figure 2). These results indicate a faster turnover of SOC in dolomitic soils, suggesting a more rapidly cycling and dynamic SOC pool. This aligns well with previous studies in alpine grassland soils at low elevation that show a faster SOC cycling at higher pH (Leifeld et al. 2009) and greater SOC stocks on siliceous bedrock (Maier et al. 2025). Similarly, the importance of underlying geology for SOC storage and dynamics has been shown for mineral soils in various studies across different soil systems (Doetterl et al. 2015; Torn et al. 1997; van der Voort et al. 2016). The observed differences in SOC cycling between bedrock types are consistent with different soil geochemical characteristics governed by bedrock type affecting C stabilization. Dolomitic soils are generally shallower (Table S1) and contain fewer pedogenic oxides (Figure 1f), as carbonate weathering leaves fewer residual reactive minerals (Musso et al. 2022), which suggests a reduced capacity for mineral‐associated SOC stabilization. In contrast, silicate weathering, particularly in gneissic soils, is associated with higher PO contents, indicative of reactive mineral surfaces that promote the formation of stable mineral‐organic associations (Kleber et al. 2021). Further support was provided by in situ soil respiration measurements and the SOC mineralization under standardized conditions (Figure 5a,b), both indicating more biodegradable SOC in dolomitic than siliceous soils, especially towards higher elevation. The likely reasons for these differences among soil types include enhanced microbial and soil enzymatic activity at higher pH values in dolomitic soils (Malik et al. 2018; Rousk et al. 2009; Weil et al. 2021) as well as a higher share of herbaceous plants (Figure S3), which produces more easily decomposable litter (Djukic et al. 2010). Together with the different mineral phases, these factors likely lead to a higher degradability, younger system ages, and lower SOC stocks in dolomitic soil systems. Further, the lower C:N ratio in dolomitic soils, indicative of a higher degree of microbial transformation (Springob and Kirchmann 2003), aligns with the faster turnover. Although all siliceous soils showed a similar mineral C saturation and soil pH, gneissic soils contained higher SOC stocks and less biodegradable SOC than amphibolitic soils (Figures 1, 2, 5). These differences might stem from variations in physical weathering, with amphibolitic rock being more resistant (Nicolussi et al. 2005), leading to higher stone content (Figure S11, Table S9) and less fine earth volume storing SOC. Furthermore, the mineral composition of amphibolite promotes higher nutrient availability (Pulla et al. 2016), which might enhance SOC decomposition rates. Further studies directly characterizing clay mineralogy and mineral surface properties would help elucidate the specific mineral properties underlying the bedrock effects observed here.

In the European Alps, siliceous to calcareous lithologies account for approximately 60% and 40% of the bedrock, respectively (Pfiffner 2014). Or results documenting bedrock dependent SOC cycling and stabilization mechanisms also suggest divergent responses upon warming and greening. Dolomitic soils, with their smaller SOC stocks and faster SOC turnover (Figures 2, 3), may respond more rapidly to warming and greening, but their propensity for long‐term SOC storage is likely more limited due to their lower C stabilization potential (Figure 5d). Moreover, enhanced microbial activity due to high pH, an already high C saturation and thus low C stabilization potential and high vulnerability to disturbances (Cannone et al. 2007) might further constrain future C sequestration. In contrast to dolomitic soils, the high mineral C stabilization potential of siliceous soils potentially allows the accumulation of new C via stable organo‐mineral‐associations (Figure 5d). However, the greater SOC stocks and longer turnover times also suggest a longer response time of siliceous high elevation systems (Figures 2, 3), further evidenced by the possible remnant legacy carbon at these sites. Overall, these findings indicate that bedrock type exerts a major influence on the future trajectories of rate and capacity of C sequestration in soils and needs to be considered in future SOC assessments. However, given the high diversity of bedrock types, especially of calcareous bedrocks with a heterogenous composition of carbonates and impurities such as clay, Al‐ and Fe oxides (Maier et al. 2025), the magnitude of bedrock‐dependent differences in SOC dynamics across the fill range of alpine bedrocks remains to be determined.

### 4.4 Limited C Sequestration in Alpine Soils Upon Greening

We found total SOC stocks showed considerable variation along the transects with an 80% increase in total SOC stocks from periglacial to grassland and forest sites (Figure 2). A greater contribution of subsoil SOC at lower elevations, reflecting deeper soil development, contributed to shaping elevational SOC stock patterns. The observed increase was not linear and best represented by a sigmoidal model (Table S4; Figure S10). The threshold aligned closely with the drop in vegetation cover from the grassland to nival zone (Figure 2) and was accompanied by an abrupt change in mean system ages (Figure 3), indicating a rapid shift in SOC dynamics at the vegetation line. Downslope transport of SOC from higher elevation soils as observed for hillslope and agricultural soils (Yoo et al. 2006; Van Oost et al. 2007) is unlikely to be a major contributor here. Contributing upslope area, which was itself unrelated to elevation or bedrock type (Table S9), did not explain additional variance in SOC beyond these two predictors (Table S13). This is consistent with evidence that vegetation cover and the establishment of dense root systems drive long‐term stabilization of periglacial and alpine slopes (Musso et al. 2020). This is further supported by largely undisturbed ∆^14^C and δ^13^C depth profiles (Figure S5), which indicate little evidence for erosional and colluvial processes. In addition, the linear decrease in soil depth and increase in stone content with increasing elevation (Figure S11, Table S9), reflecting declining soil development, cannot explain the sigmoidal shape of the SOC stocks along the elevation gradients. Similar threshold declines in SOC stocks at the vegetation line have been observed in inventory‐based studies in various nival mountain regions (Ohtsuka et al. 2008; Fuchs et al. 2015; Pascual et al. 2021; Fröjd et al. 2022; Kuhry et al. 2022). A similar threshold response has also been observed along glacier forefields, SOC cycling abruptly intensifies from unvegetated to densely vegetated sites (Smittenberg et al. 2012; Guelland et al. 2013). In contrast to these early successional soils, grassland soils showed comparable SOC stocks across 700 m in elevation along each gradient (Figure 2), while exhibiting a high variability typical for alpine soils (Hitz et al. 2002). Comparable patterns of unchanged SOC stocks along “single” elevation gradients in alpine grassland soils have been observed by Egli et al. (2008) and Wu et al. (2025), while other studies report slightly declining alpine SOC stocks with elevation (Bonfanti et al. 2025; Garcia‐Pausas et al. 2007). Moreover, from grassland to forest soils, total SOC stocks remained comparable, but forest soils stored greater amounts in the organic layer with faster turnover rates (Figures 2, 3). Our SOC stocks in siliceous forest soils compared well to stocks measured in the Swiss forest soil inventory at lower elevation (Guidi et al. 2024). In contrast, our SOC stocks in dolomitic alpine soils were only half as much as those reported for calcareous forest soils (Guidi et al. 2024), possibly reflecting the broad range of soil types grouped under this category in the inventory. Overall, the observed pattern indicates that ecosystem boundaries—here the vegetation line—play a more significant role in regulating long‐term SOC storage than temperature reflected by elevation.

One reason for abrupt SOC stock changes at vegetation boundaries but not within each system along temperature gradients might be that, within a system, increasing temperatures lead in the long term to an acceleration of both plant‐derived C inputs and outputs through microbial decomposition, which overall results in a balancing out of fluxes and similar SOC stocks. In support, our study reveals that within grassland and forest soils, both SOC turnover and C inputs increased with decreasing elevation (Figures 3, 4), whereas SOC stocks remained similar (Figure 2). Carbon stabilization potential reflected by increasing SOC:PO ratios (Figure 5d) will contribute to the enhanced SOC turnover but negligible SOC stock changes under warmer climatic conditions. However, at a vegetation boundary, additional variables are changing, such as the chemical composition of plant detritus or the ratio between above‐ and belowground inputs (Solly et al. 2017; Hagedorn et al. 2025), which alters microbial decomposition and leads to a new, different equilibrium SOC stock. At the vegetation line, we attribute the pronounced SOC stock increase to the drastic increase in C inputs into soils but only a small change in temperature‐driven decomposition, resulting in a higher SOC stock.

Our data suggest that long‐term C sequestration in alpine soils will be low and restricted to the nival and vegetation‐free periglacial zone where current C input rates are exceptionally low while soil C stabilization potentials are high (Figures 2, 3). With ongoing temperature‐driven plant shifts towards higher elevation (Rumpf et al. 2022; Steinbauer et al. 2018; Chen et al. 2011), biomass production and carbon inputs into soils are expected to increase in the nival zone. The plant‐derived and microbial metabolized decomposition products can accumulate through stabilization by “free” reactive mineral surfaces until a new equilibrium is reached and sorption sites are “saturated”, similar to proposed trajectories for low elevation agricultural soil systems (Stewart et al. 2007). In our study, we observe an eightfold increase in above and belowground biomass from the nival to the densely vegetated zone (Figures 1b,c, 2), suggesting that the increase in C input must be substantial in order to sequester C in the soil. Recent studies have shown most increase in plant productivity around a MAAT of 0.5°C (Rumpf et al. 2022), which can presently be found at approximately 2400 m a.s.l. along our transects. Our results show that up until around 2750 m a.s.l., SOC stocks remained relatively stable with elevation, suggesting only little long‐term SOC sequestration. Rather, we found that the greatest potential for long‐term SOC sequestration exists around the vegetation line with a MAAT of −2.5°C where both vegetation productivity and C inputs into soils are currently very low (Figures 1b,c, 3). Likewise, observed earlier plant phenology in alpine grasslands (Zehnder et al. 2025; Shen et al. 2022) will possibly not alter long‐term C storage in alpine soils in the lower alpine belt. The time lag between warming and the upward migration of plants and soil development (Alexander et al. 2018; Hagedorn et al. 2019; Liu et al. 2026) likely slows SOC responses to climate change although the magnitude of this effect remains uncertain. Despite studies on upward migrations of single species in the nival zone (Dentant et al. 2024; Steinbauer et al. 2018; Walther et al. 2005), quantifications of increasing plant biomass and C input with future greening are limited to grasslands (Rammig et al. 2010) and largely missing at the vegetation line, yet we argue that this zonal threshold is crucial for understanding the magnitude and pace of future C uptake and stabilization in the soil.

## 5 Conclusion

Overall, our results indicate that SOC storage in high elevation soils is likely to increase upon alpine greening induced by climate warming, with the magnitude likely depending on bedrock type. However, the non‐linear sigmoidal changes of SOC stocks with elevation suggest that soils may act as C sinks primarily at the transition between the nival and grassland zone with an abrupt increase in vegetation productivity. In contrast, within the grassland and forest zone, SOC stocks showed minor changes, while ^14^C data revealed increasing SOC turnover at lower elevation. This strongly suggests that greater C inputs under warmer climatic conditions appear to be outbalanced by higher outputs, resulting in minor SOC stock changes. Low ratios of SOC to pedogenic oxides in the forest and grassland soils indicate that they are C saturated, limiting C sequestration. In high elevation nival soils, high (old) ^14^C‐based system ages of SOC combined with a high degree of microbial transformation indicate a high degree of stabilization through interactions with reactive minerals. Dolomitic soils show smaller SOC storage than siliceous soils, possibly due to less pedogenic oxides production as well as higher microbial activity at high pH values. Although the small ratios of SOC to pedogenic oxides in nival soils suggest that they would have a high potential to sequester C, rates will be limited by the low amounts of SOC inputs from vegetation in these cold ecosystems with vegetation shifts lagging behind climate warming.

## Author Contributions

**Annegret Udke:** conceptualization, formal analysis, investigation, methodology, validation, writing – original draft, writing – review and editing. **Kyra Marty:** formal analysis, investigation, writing – review and editing. **Cédric Bührer:** formal analysis, investigation, writing – review and editing. **Luisa Minich:** formal analysis, investigation, writing – review and editing. **Michael Zehnder:** conceptualization, investigation, methodology, writing – review and editing. **Marco Griepentog:** investigation, writing – review and editing. **Sebastian Doetterl:** resources, writing – review and editing. **Negar Haghipour:** formal analysis, investigation, writing – review and editing. **Timothy Eglinton:** resources, writing – review and editing. **Christian Rixen:** conceptualization, funding acquisition, methodology, writing – review and editing. **Markus Egli:** resources, supervision, writing – review and editing. **Frank Hagedorn:** conceptualization, funding acquisition, methodology, supervision, writing – original draft, writing – review and editing.

## Funding

This work was supported by the Swiss Federal Institute for Forest, Snow and Landscape Research. Schweizerischer Nationalfonds zur Förderung der Wissenschaftlichen Forschung, 193770.

## Conflicts of Interest

The authors declare no conflicts of interest.

## Supporting information


**Figure S1:** Topographic map of study location and field sites along elevation gradients on different bedrock types in Grisons, Eastern Swiss Alps.
**Figure S2:** Vegetation cover along the alpine elevation gradients on different bedrock types.
**Figure S3:** Plant functional groups along the alpine elevation gradients on different bedrock types.
**Figure S4:** SOC depth profiles for main vegetation types along the elevation gradient.
**Figure S5:** Bulk soil ^14^C content along three elevation gradients (presented as the shift from forest to grassland to nival to vegetation‐free blockfield sites) on different bedrock types.
**Figure S6:** Bulk soil ∆^14^C and microbially respired ^14^CO2 for selected sites as well as their difference (right bottom panel).
**Figure S7:** Soil profiles in blockfields of the periglacial region.
**Figure S8:** Correlations between aboveground and belowground biomass and modeled C input rates (net C inputs into the soil).
**Figure S9:** Mean system ages, total Ca and pedogenic oxide concentrations against soil pH.
**Figure S10:** Partial effect plots of gam analysis; edf, *F*‐ and *p*‐values can be found in Table S12.
**Figure S11:** Soil thickness and stone content for different depth intervals along the elevation gradients.
**Figure S12:** Contributing upslope area plotted against elevation for each bedrock type.
**Figure S13:** Correlations between mean system age with SOC:Alpy and SOC:(Alox + Feox + Mnox) mass ratios for siliceous and dolomitic bedrock types separately.
**Table S1:** Study site overview.
**Table S2:** Air temperature references for MAAT estimation along the elevation gradients (IMIS 2023).
**Table S3:** Dominant plant species for grassland and nival sites.
**Table S4:** Model parameters and model evaluation for linear and sigmoidal models representing total SOC stocks with elevation.
**Table S5:** Lag times which were applied to buffer the atmospheric ^14^C curve prior to mean system age calculation.
**Table S6:** Laboratory incubation for ^14^C analysis of the microbially respired CO_2_.
**Table S7:** Measured soil respiration (SR) in the field.
**Table S8:** TC and TN contents in selected samples on amphibolitic and gneissic bedrock.
**Table S9:** Statistical results of linear models and linear mixed‐effect models for the presented data.
**Table S10:** Statistical analysis for a linear model explaining SOC stocks with elevation, bedrock and depth.
**Table S11:** Statistical analysis for a linear model explaining modeled C inputs with elevation, bedrock and depth.
**Table S12:** Statistical results of generalized additive models (GAM) and generalized additive mixed model (GAMM).
**Table S13:** Statistical testing of contributing upslope area on SOC stocks and radiocarbon contents.
**Table S14:** Statistical output for the categorical model testing whether SOC stocks differ by vegetation line and bedrock.

## Data Availability

The data and code that supports the findings of this study are openly available at https://www.doi.org/10.16904/envidat.807.

## References

[gcb71158-bib-0001] Adamczyk, M. , J. Rüthi , and B. Frey . 2021. “Root Exudates Increase Soil Respiration and Alter Microbial Community Structure in Alpine Permafrost and Active Layer Soils.” Environmental Microbiology 23, no. 4: 2152–2168. 10.1111/1462-2920.15383. 33393203

[gcb71158-bib-0002] Alexander, J. M. , L. Chalmandrier , J. Lenoir , et al. 2018. “Lags in the Response of Mountain Plant Communities to Climate Change.” Global Change Biology 24, no. 2: 563–579. 10.1111/gcb.13976. PMC5813787 29112781

[gcb71158-bib-0003] Bascomb, C. L. 1968. “Distribution of Pyrophosphate‐Extractable Iron and Organic Carbon in Soils of Various Groups.” Journal of Soil Science 19, no. 2: 251–268. 10.1111/j.1365-2389.1968.tb01538.x.

[gcb71158-bib-0004] Ben‐Shachar, M. S. , D. Lüdecke , and D. Makowski . 2020. “Effectsize: Estimation of Effect Size Indices and Standardized Parameters.” Journal of Open Source Software 5, no. 56: 2815. 10.21105/joss.02815.

[gcb71158-bib-0120] Bonfanti, N. , P. Choler , N. Khedim , et al. 2025. “Drivers of Soil Organic Carbon Stocks and Stability Along Elevation Gradients.” Geoderma 461: 117452. 10.1016/j.geoderma.2025.117452.

[gcb71158-bib-0005] Bright, K. , B. Dienes , M. Keiluweit , C. Rixen , and M. Aeppli . 2025. “Climate Change Impacts on Organic Carbon Cycling in European Alpine Soils.” Soil Biology and Biochemistry 210: 109891. 10.1016/j.soilbio.2025.109891.

[gcb71158-bib-0006] Budge, K. , J. Leifeld , E. Hiltbrunner , and J. Fuhrer . 2011. “Alpine Grassland Soils Contain Large Proportion of Labile Carbon but Indicate Long Turnover Times.” Biogeosciences 8, no. 7: 1911–1923. 10.5194/bg-8-1911-2011.

[gcb71158-bib-0007] Cannone, N. , S. Sgorbati , and M. Guglielmin . 2007. “Unexpected Impacts of Climate Change on Alpine Vegetation.” Frontiers in Ecology and the Environment 5, no. 7: 360–364. 10.1890/1540-9295(2007)5[360:UIOCCO]2.0.CO;2.

[gcb71158-bib-0008] Chen, I.‐C. , J. K. Hill , R. Ohlemüller , D. B. Roy , and C. D. Thomas . 2011. “Rapid Range Shifts of Species Associated With High Levels of Climate Warming.” Science 333, no. 6045: 1024–1026. 10.1126/science.1206432. 21852500

[gcb71158-bib-0009] Chen, J. , L. Elsgaard , K. J. van Groenigen , et al. 2020. “Soil Carbon Loss With Warming: New Evidence From Carbon‐Degrading Enzymes.” Global Change Biology 26, no. 4: 1944–1952. 10.1111/gcb.14986. 31909849

[gcb71158-bib-0010] Choler, P. , A. Bayle , N. Fort , and S. Gascoin . 2024. “Waning Snowfields Have Transformed Into Hotspots of Greening Within the Alpine Zone.” Nature Climate Change 15: 80–85. 10.1038/s41558-024-02177-x.

[gcb71158-bib-0011] Dahlgren, R. A. 1994. “Quantification of Allophane and Imogolite.” In Quantitative Methods in Soil Mineralogy, edited by J. E. Amonette and J. W. Stucki , 430–451. Soil Science Society of America.

[gcb71158-bib-0012] Dentant, C. , B. Z. Carlson , N. Bartalucci , A. Bayle , and S. Lavergne . 2024. “Anthropocene Trajectories of High Alpine Vegetation on Mont‐Blanc Nunataks.” Botany Letters 171, no. 1: 65–79. 10.1080/23818107.2023.2231503.

[gcb71158-bib-0013] Djukic, I. , F. Zehetner , M. Tatzber , and M. H. Gerzabek . 2010. “Soil Organic‐Matter Stocks and Characteristics Along an Alpine Elevation Gradient.” Journal of Plant Nutrition and Soil Science 173, no. 1: 30–38. 10.1002/jpln.200900027.

[gcb71158-bib-0014] Doetterl, S. , A. Stevens , J. Six , et al. 2015. “Soil Carbon Storage Controlled by Interactions Between Geochemistry and Climate.” Nature Geoscience 8, no. 10: 780–783. 10.1038/ngeo2516.

[gcb71158-bib-0015] Dümig, A. , R. Smittenberg , and I. Kögel‐Knabner . 2011. “Concurrent Evolution of Organic and Mineral Components During Initial Soil Development After Retreat of the Damma Glacier, Switzerland.” Geoderma 163, no. 1–2: 83–94. 10.1016/j.geoderma.2011.04.006.

[gcb71158-bib-0016] Egli, M. , C. Merkli , G. Sartori , A. Mirabella , and M. Plötze . 2008. “Weathering, Mineralogical Evolution and Soil Organic Matter Along a Holocene Soil Toposequence Developed on Carbonate‐Rich Materials.” Geomorphology 97, no. 3–4: 675–696. 10.1016/j.geomorph.2007.09.011.

[gcb71158-bib-0017] Elzhov, T. , K. Mullen , A. Spiess , and B. Bolker . 2023. minpack.lm: R Interface to the Levenberg‐Marquardt Nonlinear Least‐Squares Algorithm Found in MINPACK, Plus Support for Bounds. https://CRAN.R‐project.org/package=minpack.lm.

[gcb71158-bib-0018] Emmenegger, L. , M. Leuenberger , and M. Steinbacher . 2023. “ICOS ATC/CAL 14C Release, Jungfraujoch (13.9 m), 2015‐09‐21–2022‐08‐07, ICOS RI.” https://meta.icos‐cp.eu/objects/mZZZOlWKAZq‐nWd1saWzVNuG.

[gcb71158-bib-0019] Enri, S. R. , F. Petrella , F. Ungaro , et al. 2021. “Relative Importance of Plant Species Composition and Environmental Factors in Affecting Soil Carbon Stocks of Alpine Pastures (NW Italy).” Agriculture 11, no. 11: 1047. 10.3390/agriculture11111047.

[gcb71158-bib-0020] Fang, K. , S. Qin , L. Chen , Q. Zhang , and Y. Yang . 2019. “Al/Fe Mineral Controls on Soil Organic Carbon Stock Across Tibetan Alpine Grasslands.” Journal of Geophysical Research: Biogeosciences 124, no. 2: 247–259. 10.1029/2018JG004782.

[gcb71158-bib-0021] Fröjd, C. , D. Trombotto Liaudat , C. Scheer , I. Pecker Marcosig , and P. Kuhry . 2022. “Soil Organic Carbon Stocks in Mountain Periglacial Areas of Northern Patagonia (Argentina).” Arctic, Antarctic, and Alpine Research 54, no. 1: 176–199. 10.1080/15230430.2022.2062102.

[gcb71158-bib-0022] Fuchs, M. , P. Kuhry , and G. Hugelius . 2015. “Low Below‐Ground Organic Carbon Storage in a Subarctic Alpine Permafrost Environment.” Cryosphere 9, no. 2: 427–438. 10.5194/tc-9-427-2015.

[gcb71158-bib-0023] Garcia‐Pausas, J. , P. Casals , L. Camarero , et al. 2007. “Soil Organic Carbon Storage in Mountain Grasslands of the Pyrenees: Effects of Climate and Topography.” Biogeochemistry 82, no. 3: 279–289. 10.1007/s10533-007-9071-9.

[gcb71158-bib-0024] Gautschi, P. 2017. A New Method to Graphitize CO_2_ From Atmospheric Air for Radiocarbon Analysis [Master Thesis]. ETH Zurich.

[gcb71158-bib-0025] Gee, G. W. , and J. W. Bauder . 1986. “Particle‐Size Analysis.” In Methods of Soil Analysis, 383–411. John Wiley & Sons, Ltd. 10.2136/sssabookser5.1.2ed.c15.

[gcb71158-bib-0026] Gehrig‐Fasel, J. , A. Guisan , and N. E. Zimmermann . 2007. “Tree Line Shifts in the Swiss Alps: Climate Change or Land Abandonment?” Journal of Vegetation Science 18, no. 4: 571–582. 10.1111/j.1654-1103.2007.tb02571.x.

[gcb71158-bib-0027] Georgiou, K. , D. Angers , R. E. Champiny , et al. 2025. “Soil Carbon Saturation: What Do we Really Know?” Global Change Biology 31, no. 5: e70197. 10.1111/gcb.70197. PMC12062940 40345163

[gcb71158-bib-0028] Grant, K. E. , R. G. Hilton , and V. V. Galy . 2023. “Global Patterns of Radiocarbon Depletion in Subsoil Linked to Rock‐Derived Organic Carbon.” Geochemical Perspectives Letters 25, no. 25: 36–40.

[gcb71158-bib-0029] Guelland, K. , J. Esperschütz , D. Bornhauser , S. M. Bernasconi , R. Kretzschmar , and F. Hagedorn . 2013. “Mineralisation and Leaching of C From 13C Labelled Plant Litter Along an Initial Soil Chronosequence of a Glacier Forefield.” Soil Biology and Biochemistry 57: 237–247. 10.1016/j.soilbio.2012.07.002.

[gcb71158-bib-0030] Guidi, C. , S. Zimmermann , L. Walthert , M. Didion , S. Gosheva‐Oney , and F. Hagedorn . 2024. “Soil Organic Carbon Drivers in the Swiss Forest Soil Inventory [Dataset].” *EnviDat*. https://www.envidat.ch/dataset/a8e75c2f‐bce8‐4311‐8867‐4b48a6b4f2ff.

[gcb71158-bib-0031] Hagedorn, F. , K. Gavazov , and J. M. Alexander . 2019. “Above‐ and Belowground Linkages Shape Responses of Mountain Vegetation to Climate Change.” Science 365, no. 6458: 1119–1123. 10.1126/science.aax4737. 31515385

[gcb71158-bib-0032] Hagedorn, F. , J. Imboden , P. A. Moiseev , et al. 2025. “Distinct Changes in Carbon, Nitrogen, and Phosphorus Cycling in the Litter Layer Across Two Contrasting Forest–Tundra Ecotones.” Biogeosciences 22, no. 12: 2959–2977. 10.5194/bg-22-2959-2025.

[gcb71158-bib-0033] Hagedorn, F. , M. Martin , C. Rixen , et al. 2010. “Short‐Term Responses of Ecosystem Carbon Fluxes to Experimental Soil Warming at the Swiss Alpine Treeline.” Biogeochemistry 97, no. 1: 7–19. 10.1007/s10533-009-9297-9.

[gcb71158-bib-0034] Hitz, C. , M. Egli , and P. Fitze . 2002. “Determination of Sampling Volumes Needed for Representative Analysis of Alpine Soils.” Journal of Plant Nutrition and Soil Science 165, no. 3: 326–331.

[gcb71158-bib-0035] Hua, Q. , J. C. Turnbull , G. M. Santos , et al. 2022. “Atmospheric Radiocarbon for the Period 1950–2019.” Radiocarbon 64, no. 4: 723–745. 10.1017/RDC.2021.95.

[gcb71158-bib-0036] Intercantonal Measurement and Information System IMIS . 2023. “IMIS Measuring Network.” *EnviDat*. 10.16904/envidat.406.

[gcb71158-bib-0037] Jia, N. , L. Li , H. Guo , and M. Xie . 2024. “Important Role of Fe Oxides in Global Soil Carbon Stabilization and Stocks.” Nature Communications 15, no. 1: 10318. 10.1038/s41467-024-54832-8. PMC11605054 39609446

[gcb71158-bib-0038] Jiménez, J. J. , and L. Villar . 2017. “Mineral Controls on Soil Organic C Stabilization in Alpine and Subalpine Soils in the Central Pyrenees: Insights From Wet Oxidation Methods, Mineral Dissolution Treatment and Radiocarbon Dating.” Catena 149: 363–373. 10.1016/j.catena.2016.10.011.

[gcb71158-bib-0039] Kaiser, M. , D. P. Zederer , R. H. Ellerbrock , M. Sommer , and B. Ludwig . 2016. “Effects of Mineral Characteristics on Content, Composition, and Stability of Organic Matter Fractions Separated From Seven Forest Topsoils of Different Pedogenesis.” Geoderma 263: 1–7.

[gcb71158-bib-0040] Kammer, A. , F. Hagedorn , I. Shevchenko , et al. 2009. “Treeline Shifts in the Ural Mountains Affect Soil Organic Matter Dynamics.” Global Change Biology 15, no. 6: 1570–1583. 10.1111/j.1365-2486.2009.01856.x.

[gcb71158-bib-0041] Khandakar, T. , C. Guppy , S. M. F. Rabbi , and H. Daniel . 2021. “Poorly Crystalline Iron and Aluminium Oxides Contribute to the Carbon Saturation and Sorption of Dissolved Organic Carbon in the Soil.” Soil Use and Management 37, no. 1: 120–125. 10.1111/sum.12662.

[gcb71158-bib-0042] Kleber, M. , I. C. Bourg , E. K. Coward , C. M. Hansel , S. C. B. Myneni , and N. Nunan . 2021. “Dynamic Interactions at the Mineral–Organic Matter Interface.” Nature Reviews Earth and Environment 2, no. 6: 402–421. 10.1038/s43017-021-00162-y.

[gcb71158-bib-0043] Körner, C. 2003. “Alpine Soils.” In Alpine Plant Life–Functional Plant Ecology of High Mountain Ecosystems, edited by C. Körner , 2nd ed., 63–76. Springer. 10.1007/978-3-642-18970-8.

[gcb71158-bib-0044] Kuhry, P. , E. Makopoulou , D. Pascual Descarrega , I. Pecker Marcosig , and D. Trombotto Liaudat . 2022. “Soil Organic Carbon Stocks in the High Mountain Permafrost Zone of the Semi‐Arid Central Andes (Cordillera Frontal, Argentina).” Catena 217: 106434. 10.1016/j.catena.2022.106434.

[gcb71158-bib-0045] Kuznetsova, A. , P. Brockhoff , and R. Christensen . 2017. “lmerTest Package: Tests in Linear Mixed Effects Models.” Journal of Statistical Software 12: 13. 10.18637/jss.v082.i13.

[gcb71158-bib-0046] Lavallee, J. M. , J. L. Soong , and M. F. Cotrufo . 2020. “Conceptualizing Soil Organic Matter Into Particulate and Mineral‐Associated Forms to Address Global Change in the 21st Century.” Global Change Biology 26, no. 1: 261–273. 10.1111/gcb.14859. 31587451

[gcb71158-bib-0047] Lehmann, J. , and M. Kleber . 2015. “The Contentious Nature of Soil Organic Matter.” Nature Perspective 12: 6069. 10.1038/nature16069. 26595271

[gcb71158-bib-0048] Leifeld, J. , S. Meyer , K. Budge , M. T. Sebastia , M. Zimmermann , and J. Fuhrer . 2015. “Turnover of Grassland Roots in Mountain Ecosystems Revealed by Their Radiocarbon Signature: Role of Temperature and Management.” PLoS One 10, no. 3: e0119184. 10.1371/journal.pone.0119184. PMC4347979 25734640

[gcb71158-bib-0049] Leifeld, J. , M. Zimmermann , J. Fuhrer , and F. Conen . 2009. “Storage and Turnover of Carbon in Grassland Soils Along an Elevation Gradient in the Swiss Alps.” Global Change Biology 15, no. 3: 668–679. 10.1111/j.1365-2486.2008.01782.x.

[gcb71158-bib-0050] Leonelli, G. , M. Pelfini , U. Morra di Cella , and V. Garavaglia . 2011. “Climate Warming and the Recent Treeline Shift in the European Alps: The Role of Geomorphological Factors in High‐Altitude Sites.” Ambio 40, no. 3: 264–273. 10.1007/s13280-010-0096-2. PMC3357808 21644455

[gcb71158-bib-0117] Liu, L. , K. Zhu , J. Chen , et al. 2026. “Shifts in the Upper Limit of Alpine Grasslands Lag Behind Climate Warming Across the Tibetan Plateau.” Global Change Biology 32, no. 3: e70820. 10.1111/gcb.70820. 41873643

[gcb71158-bib-0051] Loeppert, R. H. , and W. P. Inskeep . 1996. “Iron.” In Methods of Soil Analysis. Part 3. Chemical Methods, edited by D. L. Sparks , A. L. Page , P. A. Helmke , et al., 639–664. Soil Science Society of America, American Society of Agronomy. 10.2136/sssabookser5.3.c23.

[gcb71158-bib-0116] Ma, Z. , Y. Chen , W. Xu , and M. Liu . 2022. “Increased Plant Growth May Offset Soil Carbon Loss Caused by Warming in an Alpine *Sibiraea angustata* Shrub Ecosystem on the Eastern Qinghai‐Tibet Plateau.” Ecological Indicators 141: 109066. 10.1016/j.ecolind.2022.109066.

[gcb71158-bib-0052] Maggetti, M. , and M. Flisch . 1993. “Evolution of the Silvretta Nappe.” In Pre‐Mesozoic Geology in the Alps, edited by J. F. von Raumer and F. Neubauer , 469–484. Springer. 10.1007/978-3-642-84640-3_28.

[gcb71158-bib-0053] Maier, A. , M. E. Macfarlane , M. Griepentrog , and S. Doetterl . 2025. “Parent Material Geochemistry – And Not Plant Biomass – As the Key Factor Shaping Soil Organic Carbon Stocks in European Alpine Grasslands.” Biogeosciences 22, no. 22: 7337–7361. 10.5194/bg-22-7337-2025.

[gcb71158-bib-0054] Malik, A. A. , J. Puissant , K. M. Buckeridge , et al. 2018. “Land Use Driven Change in Soil pH Affects Microbial Carbon Cycling Processes.” Nature Communications 9, no. 1: 3591. 10.1038/s41467-018-05980-1. PMC6123395 30181597

[gcb71158-bib-0055] Mietkiewicz, N. , D. Kulakowski , J. Rogan , and P. Bebi . 2017. “Long‐Term Change in Sub‐Alpine Forest Cover, Tree Line and Species Composition in the Swiss Alps.” Journal of Vegetation Science 28, no. 5: 951–964. 10.1111/jvs.12561.

[gcb71158-bib-0056] Minich, L. I. , D. Geissbühler , S. Tobler , et al. 2026. “Conceptualising Carbon Cycling Pathways Across Different Land‐Use Types Based on Rates and Ages of Soil‐Respired CO_2_ .” Biogeosciences 23, no. 2: 811–829. 10.5194/bg-23-811-2026.

[gcb71158-bib-0057] Moreno‐Duborgel, M. , S. Gosheva‐Oney , B. González‐Domínguez , et al. 2025. “Shifting Carbon Fractions in Forest Soils Offset ^14^C‐Based Turnover Times Along a 1700 m Elevation Gradient.” Global Change Biology 31, no. 7: e70326. 10.1111/gcb.70326. PMC12254928 40650491

[gcb71158-bib-0058] Musso, A. , M. E. Ketterer , K. Greinwald , C. Geitner , and M. Egli . 2020. “Rapid Decrease of Soil Erosion Rates With Soil Formation and Vegetation Development in Periglacial Areas.” Earth Surface Processes and Landforms 45, no. 12: 2824–2839. 10.1002/esp.4932.

[gcb71158-bib-0059] Musso, A. , D. Tikhomirov , M. L. Plötze , et al. 2022. “Soil Formation and Mass Redistribution During the Holocene Using Meteoric10 be, Soil Chemistry and Mineralogy.” Geosciences 12, no. 2: 99. 10.3390/geosciences12020099.

[gcb71158-bib-0060] Nicolussi, K. , M. Kaufmann , G. Patzelt , J. Plicht Van Der , and A. Thurner . 2005. “Holocene Tree‐Line Variability in the Kauner Valley, Central Eastern Alps, Indicated by Dendrochronological Analysis of Living Trees and Subfossil Logs.” Vegetation History and Archaeobotany 14, no. 3: 221–234. 10.1007/s00334-005-0013-y.

[gcb71158-bib-0061] Nogués‐Bravo, D. , M. B. Araújo , M. P. Errea , and J. P. Martínez‐Rica . 2007. “Exposure of Global Mountain Systems to Climate Warming During the 21st Century.” Global Environmental Change 17, no. 3: 420–428. 10.1016/j.gloenvcha.2006.11.007.

[gcb71158-bib-0119] Ohtsuka, T. , M. Hirota , X. Zhang , et al. 2008. “Soil Organic Carbon Pools in Alpine to Nival Zones Along an Altitudinal Gradient (4400–5300 m) on the Tibetan Plateau.” Polar Science 2, no. 4: 277–285. 10.1016/j.polar.2008.08.003.

[gcb71158-bib-0062] Pascual, D. , P. Kuhry , and T. Raudina . 2021. “Soil Organic Carbon Storage in a Mountain Permafrost Area of Central Asia (High Altai, Russia).” Ambio 50, no. 11: 2022–2037. 10.1007/s13280-020-01433-6. PMC8497679 33289052

[gcb71158-bib-0063] Pepin, N. , R. S. Bradley , H. F. Diaz , et al. 2015. “Elevation‐Dependent Warming in Mountain Regions of the World.” Nature Climate Change 5, no. 5: 424–430. 10.1038/nclimate2563.

[gcb71158-bib-0064] Pepin, N. C. , E. Arnone , A. Gobiet , et al. 2022. “Climate Changes and Their Elevational Patterns in the Mountains of the World.” Reviews of Geophysics 60, no. 1: 730. 10.1029/2020RG000730.

[gcb71158-bib-0118] Pfiffner, A. 2014. Geology of the Alps (2. Auflage). John Wiley & Sons. (Original work published 2014, Haupt Berne).

[gcb71158-bib-0065] Pintaldi, E. , M. E. D'Amico , N. Colombo , et al. 2021. “Hidden Soils and Their Carbon Stocks at High‐Elevation in the European Alps (North‐West Italy).” Catena 198: 105044. 10.1016/j.catena.2020.105044.

[gcb71158-bib-0066] Poeplau, C. , C. Vos , and A. Don . 2017. “Soil Organic Carbon Stocks Are Systematically Overestimated by Misuse of the Parameters Bulk Density and Rock Fragment Content.” Soil 3, no. 1: 61–66. 10.5194/soil-3-61-2017.

[gcb71158-bib-0067] Porras, R. C. , C. E. Hicks Pries , K. J. McFarlane , P. J. Hanson , and M. S. Torn . 2017. “Association With Pedogenic Iron and Aluminium: Effects on Soil Organic Carbon Storage and Stability in Four Temperate Forest Soils.” Biogeochemistry 133, no. 3: 333–345. 10.1007/s10533-017-0337-6.

[gcb71158-bib-0068] Prietzel, J. , and M. Wiesmeier . 2019. “A Concept to Optimize the Accuracy of Soil Surface Area and SOC Stock Quantification in Mountainous Landscapes.” Geoderma 356: 113922. 10.1016/j.geoderma.2019.113922.

[gcb71158-bib-0069] Puissant, J. , R. T. E. Mills , B. J. M. Robroek , et al. 2017. “Climate Change Effects on the Stability and Chemistry of Soil Organic Carbon Pools in a Subalpine Grassland.” Biogeochemistry 132, no. 1: 123–139. 10.1007/s10533-016-0291-8.

[gcb71158-bib-0070] Pulla, S. , J. Riotte , H. S. Suresh , H. S. Dattaraja , and R. Sukumar . 2016. “Controls of Soil Spatial Variability in a Dry Tropical Forest.” PLoS One 11, no. 4: e0153212. 10.1371/journal.pone.0153212. PMC4839752 27100088

[gcb71158-bib-0071] R Core Team . 2025. R: A Language and Environment for Statistical Computing. R Foundation for Statistical Computing.

[gcb71158-bib-0072] Rammig, A. , T. Jonas , N. E. Zimmermann , and C. Rixen . 2010. “Changes in Alpine Plant Growth Under Future Climate Conditions.” Biogeosciences 7, no. 6: 2013–2024. 10.5194/bg-7-2013-2010.

[gcb71158-bib-0073] Rangwala, I. , E. Sinsky , and J. R. Miller . 2013. “Amplified Warming Projections for High Altitude Regions of the Northern Hemisphere Mid‐Latitudes From CMIP5 Models.” Environmental Research Letters 8, no. 2: 24040. 10.1088/1748-9326/8/2/024040.

[gcb71158-bib-0074] Rasmussen, C. , M. S. Torn , and R. J. Southard . 2005. “Mineral Assemblage and Aggregates Control Carbon Dynamics in a California Conifer Forest.” Soil Science Society of America Journal 69, no. 6: 1711–1721. 10.2136/sssaj2005.0040.

[gcb71158-bib-0075] Rousk, J. , P. C. Brookes , and E. Bååth . 2009. “Contrasting Soil pH Effects on Fungal and Bacterial Growth Suggest Functional Redundancy in Carbon Mineralization.” Applied and Environmental Microbiology 75, no. 6: 1589–1596. 10.1128/AEM.02775-08. PMC2655475 19151179

[gcb71158-bib-0076] Rowley, M. C. , S. Grand , and É. P. Verrecchia . 2018. “Calcium‐Mediated Stabilisation of Soil Organic Carbon.” Biogeochemistry 137, no. 1: 27–49. 10.1007/s10533-017-0410-1.

[gcb71158-bib-0077] Ruff, M. , S. Fahrni , H. W. Gäggeler , et al. 2010. “On‐Line Radiocarbon Measurements of Small Samples Using Elemental Analyzer and MICADAS Gas Ion Source.” Radiocarbon 52, no. 4: 1645–1656. 10.1017/S003382220005637X.

[gcb71158-bib-0078] Ruiz, F. , T. O. Ferreira , G. C. Delghingaro , V. S. de Souza Júnior , J. A. M. Demattê , and X. L. Otero . 2026. “Evaluating Sodium Pyrophosphate Extraction to Probe Soil Organo‐Mineral Interactions Across Contrasting Pedogenic Stages.” European Journal of Soil Science 77, no. 2: e70330. 10.1111/ejss.70330.

[gcb71158-bib-0079] Rumpf, S. B. , M. Gravey , O. Brönnimann , et al. 2022. “From White to Green: Snow Cover Loss and Increased Vegetation Productivity in the European Alps.” Science 376: 1119–1122. 10.1126/science.abn6697 35653482

[gcb71158-bib-0080] Shen, M. , S. Wang , N. Jiang , et al. 2022. “Plant Phenology Changes and Drivers on the Qinghai–Tibetan Plateau.” Nature Reviews Earth & Environment 3, no. 10: 633–651. 10.1038/s43017-022-00317-5.

[gcb71158-bib-0081] Sierra, C. A. , M. Müller , H. Metzler , S. Manzoni , and S. E. Trumbore . 2017. “The Muddle of Ages, Turnover, Transit, and Residence Times in the Carbon Cycle.” Global Change Biology 23, no. 5: 1763–1773. 10.1111/gcb.13556. 27886430

[gcb71158-bib-0082] Sierra, C. A. , M. Müller , and S. E. Trumbore . 2014. “Modeling Radiocarbon Dynamics in Soils: SoilR Version 1.1.” Geoscientific Model Development 7, no. 5: 1919–1931. 10.5194/gmd-7-1919-2014.

[gcb71158-bib-0084] Smittenberg, R. H. , M. Gierga , H. Göransson , I. Christl , D. Farinotti , and S. M. Bernasconi . 2012. “Climate‐Sensitive Ecosystem Carbon Dynamics Along the Soil Chronosequence of the Damma Glacier Forefield, Switzerland.” Global Change Biology 18, no. 6: 1941–1955. 10.1111/j.1365-2486.2012.02654.x.

[gcb71158-bib-0085] Solly, E. , I. Schöning , S. Boch , et al. 2013. “Mean Age of Carbon in Fine Roots From Temperate Forests and Grasslands With Different Management.” Biogeosciences 10, no. 7: 4833–4843. 10.5194/bg-10-4833-2013.

[gcb71158-bib-0086] Solly, E. F. , I. Djukic , P. A. Moiseev , et al. 2017. “Treeline Advances and Associated Shifts in the Ground Vegetation Alter Fine Root Dynamics and Mycelia Production in the South and Polar Urals.” Oecologia 183, no. 2: 571–586. 10.1007/s00442-016-3785-0. 27904966

[gcb71158-bib-0087] Springob, G. , and H. Kirchmann . 2003. “Bulk Soil C to N Ratio as a Simple Measure of Net N Mineralization From Stabilized Soil Organic Matter in Sandy Arable Soils.” Soil Biology & Biochemistry 35, no. 4: 629–632. 10.1016/S0038-0717(03)00052-X.

[gcb71158-bib-0088] Steinbauer, M. J. , J.‐A. Grytnes , G. Jurasinski , et al. 2018. “Accelerated Increase in Plant Species Richness on Mountain Summits Is Linked to Warming.” Nature 556: 231–234. 10.1038/s41586-018-0005-6 29618821

[gcb71158-bib-0089] Stewart, C. E. , K. Paustian , R. T. Conant , A. F. Plante , and J. Six . 2007. “Soil Carbon Saturation: Concept, Evidence and Evaluation.” Biogeochemistry 86, no. 1: 19–31. 10.1007/s10533-007-9140-0.

[gcb71158-bib-0090] Streit, K. , F. Hagedorn , D. Hiltbrunner , et al. 2014. “Soil Warming Alters Microbial Substrate Use in Alpine Soils.” Global Change Biology 20, no. 4: 1327–1338. 10.1111/gcb.12396. 24106016

[gcb71158-bib-0091] Stuiver, M. , and H. A. Polach . 1977. “Discussion Reporting of ^14^C Data.” Radiocarbon 19, no. 3: 355–363. 10.1017/S0033822200003672.

[gcb71158-bib-0092] SwissMeteo Bundesamt für Meteorologie und Klimatologie MeteoSchweiz . 2024. Normwert‐Gitter Niederschlag Rnorm9120. SwissMeteo.

[gcb71158-bib-0093] SwissTopo Bundesamt für Landestopografie Schweiz, Federal Office of Topography Switzerland . 2019. swissALTI3D [Dataset]. SwissTopo.

[gcb71158-bib-0094] SwissTopo Bundesamt für Landestopografie Schweiz, Federal Office of Topography Switzerland . 2024. Geological Map of Switzerland 1:500.000 ‐ GeoKarten 500 Vektor [Data Set]. SwissTopo.

[gcb71158-bib-0095] Synal, H.‐A. , M. Stocker , and M. Suter . 2007. “MICADAS: A New Compact Radiocarbon AMS System.” Nuclear Instruments and Methods in Physics Research Section B: Beam Interactions With Materials and Atoms 259, no. 1: 7–13. 10.1016/j.nimb.2007.01.138.

[gcb71158-bib-0096] Torn, M. S. , S. E. Trumbore , O. A. Chadwick , P. M. Vitousek , and D. M. Hendricks . 1997. “Mineral Control of Soil Organic Carbon Storage and Turnover.” Nature 389, no. 6647: 170–173. 10.1038/38260.

[gcb71158-bib-0097] Trumbore, S. 2000. “Age of Soil Organic Matter and Soil Respiration: Radiocarbon Constraints on Belowground C Dynamics.” Ecological Applications 10, no. 2: 399–411. 10.1890/1051-0761(2000)010[0399:AOSOMA]2.0.CO;2.

[gcb71158-bib-0098] Trumbore, S. 2009. “Radiocarbon and Soil Carbon Dynamics.” Annual Review of Earth and Planetary Sciences 37: 47–66. 10.1146/annurev.earth.36.031207.124300.

[gcb71158-bib-0099] Trumbore, S. E. , O. A. Chadwick , and R. Amundson . 1996. “Rapid Exchange Between Soil Carbon and Atmospheric Carbon Dioxide Driven by Temperature Change.” Science 272, no. 5260: 393–396. 10.1126/science.272.5260.393.

[gcb71158-bib-0100] van der Voort, T. S. , F. Hagedorn , C. McIntyre , et al. 2016. “Variability in ^14^C Contents of Soil Organic Matter at the Plot and Regional Scale Across Climatic and Geologic Gradients.” Biogeosciences 13, no. 11: 3427–3439. 10.5194/bg-13-3427-2016.

[gcb71158-bib-0101] Van Oost, K. , T. A. Quine , G. Govers , et al. 2007. “The Impact of Agricultural Soil Erosion on the Global Carbon Cycle.” Science 318, no. 5850: 626–629. 10.1126/science.1145724. 17962559

[gcb71158-bib-0102] Wacker, L. , G. Bonani , M. Friedrich , et al. 2010. “MICADAS: Routine and High‐Precision Radiocarbon Dating.” Radiocarbon 52, no. 2: 252–262. 10.1017/S0033822200045288.

[gcb71158-bib-0103] Wacker, L. , M. Němec , and J. Bourquin . 2010. “A Revolutionary Graphitisation System: Fully Automated, Compact and Simple.” Nuclear Instruments & Methods in Physics Research. Section B, Beam Interactions With Materials and Atoms 268, no. 7: 931–934. 10.1016/j.nimb.2009.10.067.

[gcb71158-bib-0104] Walther, G.‐R. , S. Beißner , and C. A. Burga . 2005. “Trends in the Upward Shift of Alpine Plants.” Journal of Vegetation Science 16, no. 5: 541–548. 10.1111/j.1654-1103.2005.tb02394.x.

[gcb71158-bib-0105] Walthert, L. , U. Graf , A. Kammer , et al. 2010. “Determination of Organic and Inorganic Carbon, δ^13^C, and Nitrogen in Soils Containing Carbonates After Acid Fumigation With HCl.” Journal of Plant Nutrition and Soil Science 173, no. 2: 207–216. 10.1002/jpln.200900158.

[gcb71158-bib-0106] Weil, S.‐S. , C. Martinez‐Almoyna , G. Piton , et al. 2021. “Strong Links Between Plant Traits and Microbial Activities but Different Abiotic Drivers in Mountain Grasslands.” Journal of Biogeography 48, no. 11: 2755–2770. 10.1111/jbi.14235.

[gcb71158-bib-0107] Wood, S. , and F. Scheipl . 2025. “gamm4: Generalized Additive Mixed Models Using ‘mgcv’ and ‘lme4’.” 10.32614/CRAN.package.gamm4.

[gcb71158-bib-0108] Wood, S. N. 2011. “Fast Stable Restricted Maximum Likelihood and Marginal Likelihood Estimation of Semiparametric Generalized Linear Models.” Journal of the Royal Statistical Society. Series B, Methodological 73, no. 1: 3–36. 10.1111/j.1467-9868.2010.00749.x.

[gcb71158-bib-0109] Wu, Y. , J. Peñuelas , M. Deng , et al. 2025. “Elevational Changes in Vegetation and Soil Geochemistry Drive Thresholds in Bulk Soil Carbon and Its Key Fractions.” Journal of Ecology 113, no. 8: 1985–1996. 10.1111/1365-2745.70055.

[gcb71158-bib-0110] Yan, Y. , S. Niu , Y. He , et al. 2022. “Changing Plant Species Composition and Richness Benefit Soil Carbon Sequestration Under Climate Warming.” Functional Ecology 36, no. 12: 2906–2916. 10.1111/1365-2435.14218.

[gcb71158-bib-0111] Yang, Y. , J. Fang , Y. Tang , et al. 2008. “Storage, Patterns and Controls of Soil Organic Carbon in the Tibetan Grasslands.” Global Change Biology 14, no. 7: 1592–1599. 10.1111/j.1365-2486.2008.01591.x.

[gcb71158-bib-0112] Yoo, K. , R. Amundson , A. M. Heimsath , and W. E. Dietrich . 2006. “Spatial Patterns of Soil Organic Carbon on Hillslopes: Integrating Geomorphic Processes and the Biological C Cycle.” Geoderma 130, no. 1: 47–65. 10.1016/j.geoderma.2005.01.008.

[gcb71158-bib-0113] Zehnder, M. , B. Pfund , J. Svoboda , et al. 2025. “Snow Height Sensors Reveal Phenological Advance in Alpine Grasslands.” Global Change Biology 31, no. 5: e70195. 10.1111/gcb.70195. 40346003

[gcb71158-bib-0114] Zhao, X. , Y. Yang , H. Shen , X. Geng , and J. Fang . 2019. “Global Soil–Climate–Biome Diagram: Linking Surface Soil Properties to Climate and Biota.” Biogeosciences 16, no. 14: 2857–2871. 10.5194/bg-16-2857-2019.

[gcb71158-bib-0115] Zhou, Z. , C. Ren , C. Wang , et al. 2024. “Global Turnover of Soil Mineral‐Associated and Particulate Organic Carbon.” Nature Communications 15, no. 1: 5329. 10.1038/s41467-024-49743-7. PMC11193739 38909059

