# Supplementary material for: Non‐Linear Increases of Soil Carbon Stocks and Turnover Along Alpine Greening Gradients on Different Bedrocks
Source: Glob Chang Biol. 2026 Oct 22;32(10):e71158. doi: 10.1111/gcb.71158 (PMC13687915; doi:10.1111/gcb.71158)
Supplement: Supplementary file 1 [file GCB-32-e71158-s001.pdf]

Supplement information: Non-linear increases of soil carbon stocks and turnover along alpine greening gradients on different bedrocks

Annegret Udke[1,2], Kyra Marty[1,3], Cédric Bührer[1,2], Luisa Minich[1,4], Michael Zehnder[3,5,6], Marco Griepentog[3], Sebastian Doetterl[3], Negar Haghipour[4], Timothy Eglinton[4], Christian Rixen[5,6], Markus Egli[2], Frank Hagedorn[1]

1 – Swiss Federal Institute for Forest, Snow and Landscape Research WSL, Birmensdorf, Switzerland

2 – Department of Geography, University of Zurich, Zurich, Switzerland

3 – Department of Environmental Systems Sciences, ETH Zurich, Switzerland

4 – Department of Earth and Planetary Sciences, ETH Zurich, Zurich, Switzerland

5 – WSL Institute for Snow and Avalanche Research SLF, Davos, Switzerland

6 – Climate Change, Extremes and Natural Hazards in Alpine Regions Research Centre (CERC), Davos, Switzerland

Contact information for corresponding author(s): annegret.udke@wsl.ch

Figures

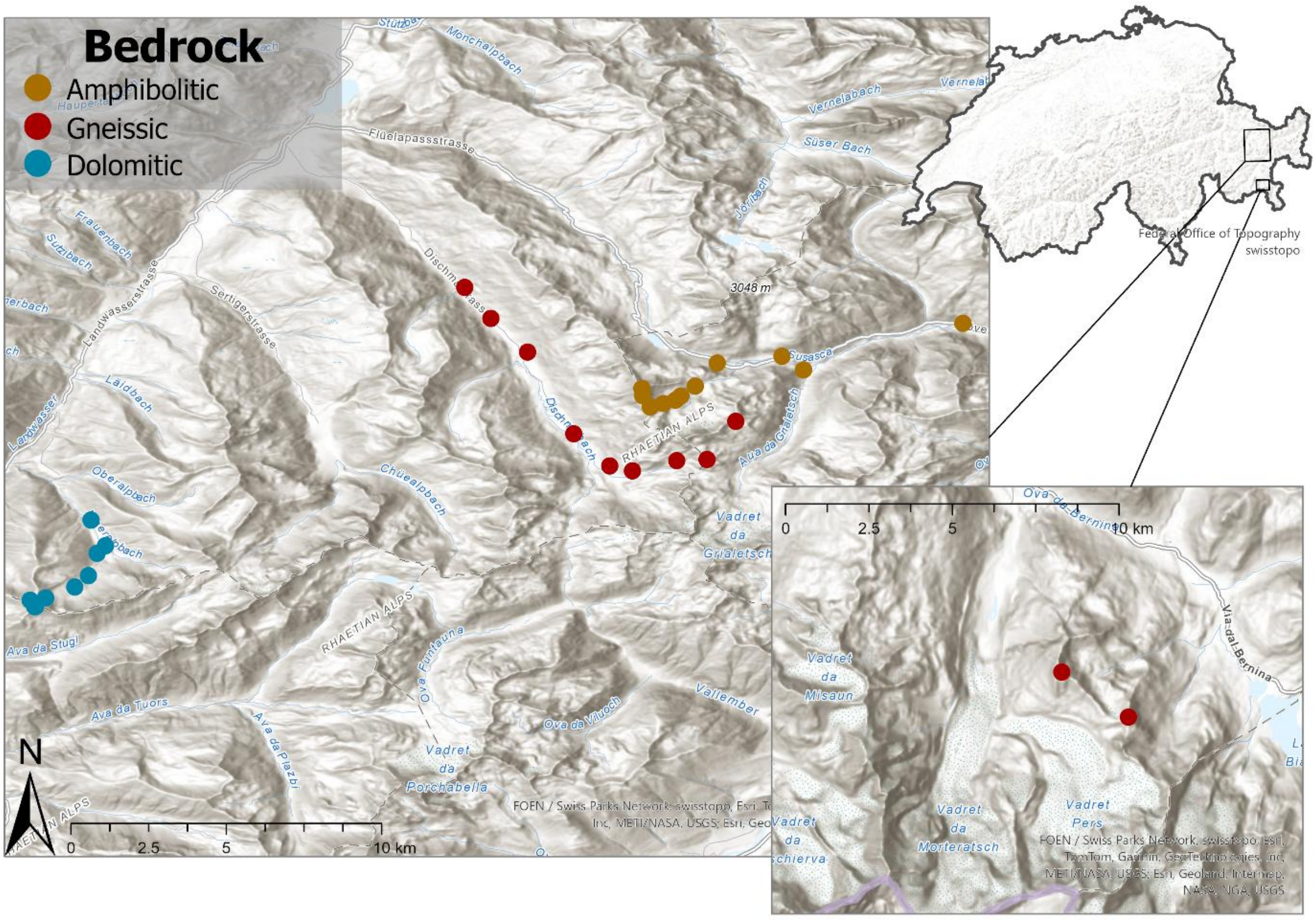

Figure S1: Topographic map of study location and field sites along elevation gradients on different bedrock types in Grisons, Eastern Swiss Alps

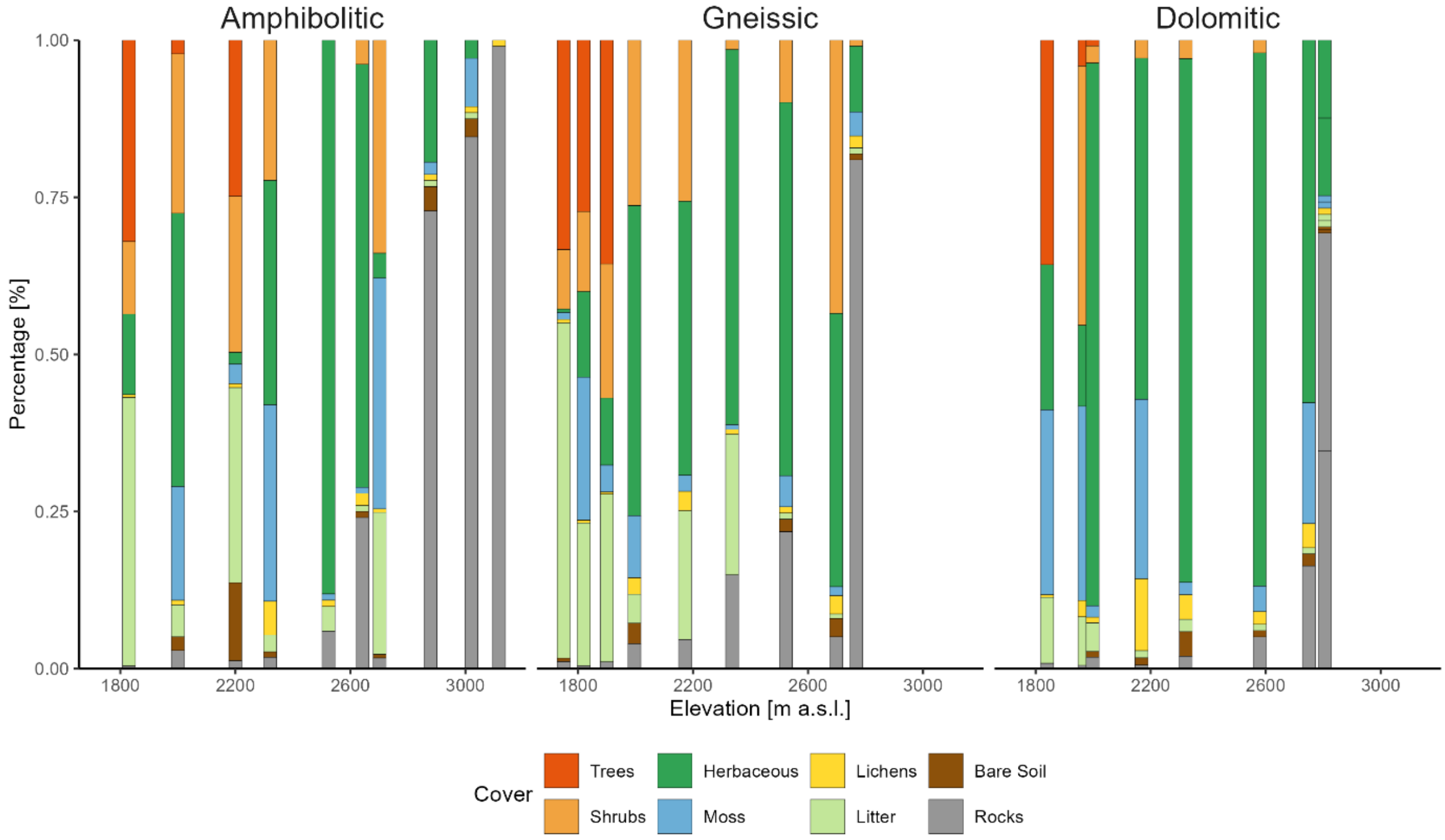

Figure S2: Vegetation cover along the alpine elevation gradients on different bedrock types

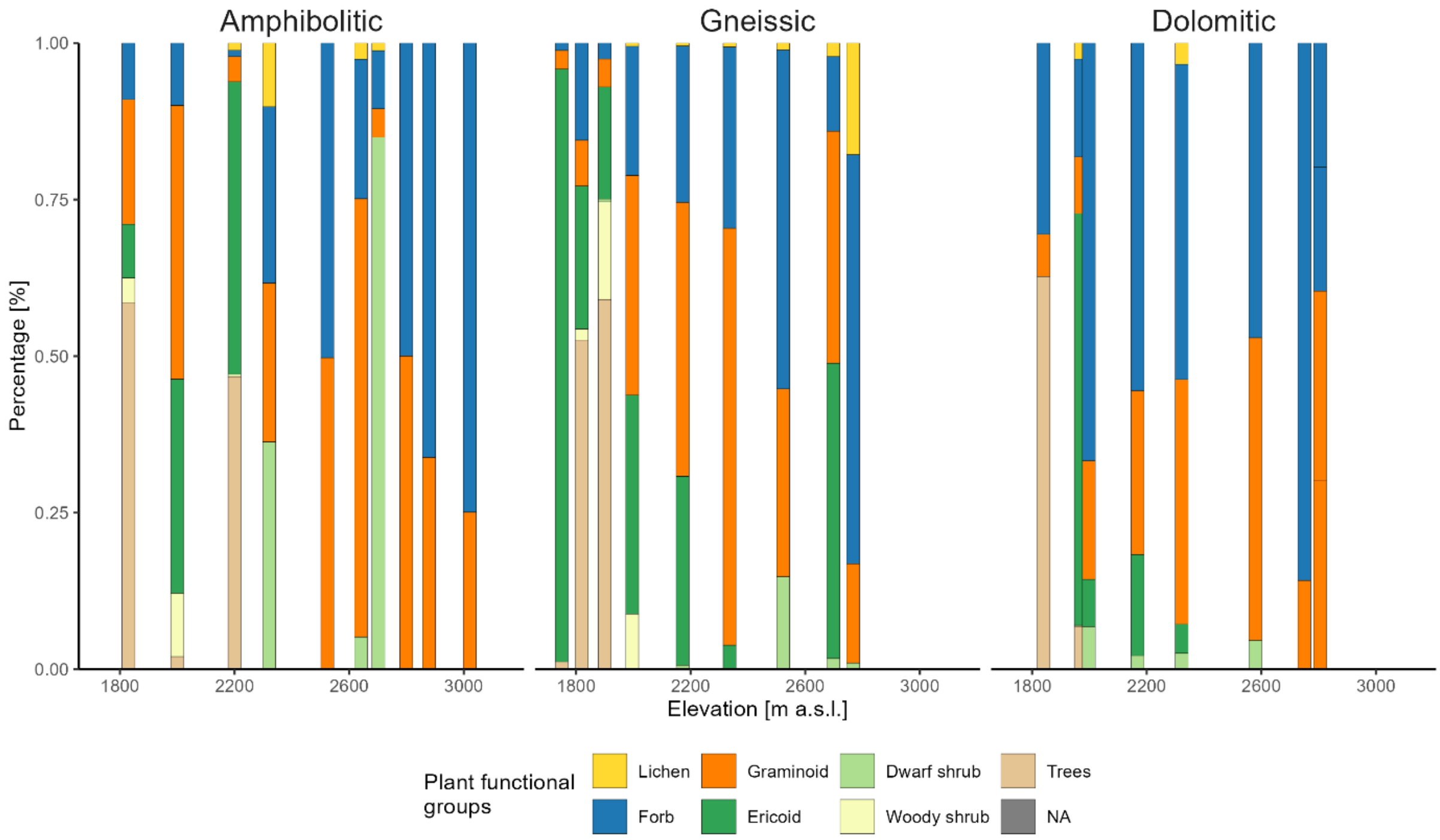

Figure S3: Plant functional groups along the alpine elevation gradients on different bedrock types

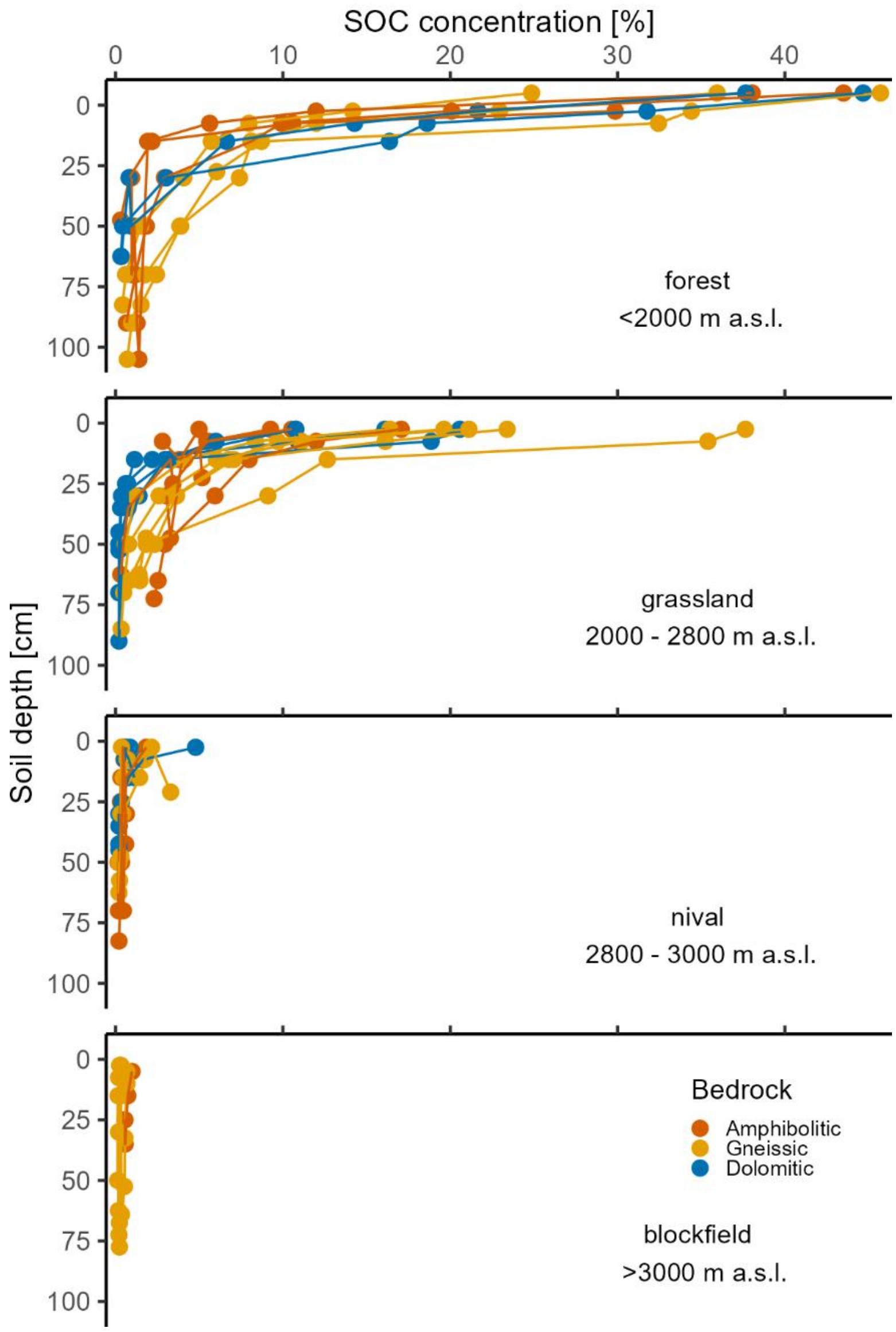

Figure S4: SOC depth profiles for main vegetation types along the elevation gradient

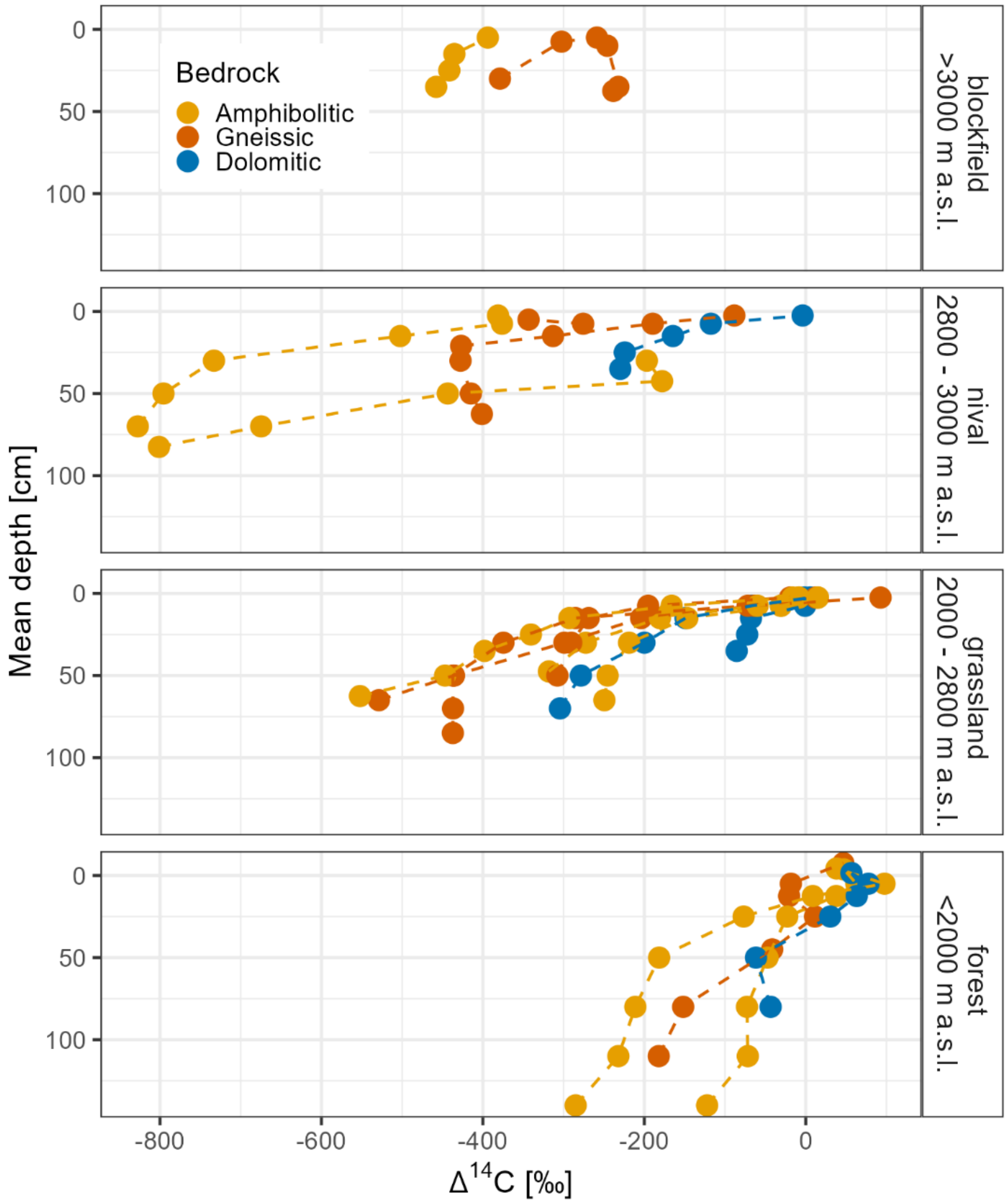

Figure S5: Bulk soil ^{14}C content along three elevation gradients (presented as the shift from forest to grassland to nival to vegetation-free blockfield sites) on different bedrock types.

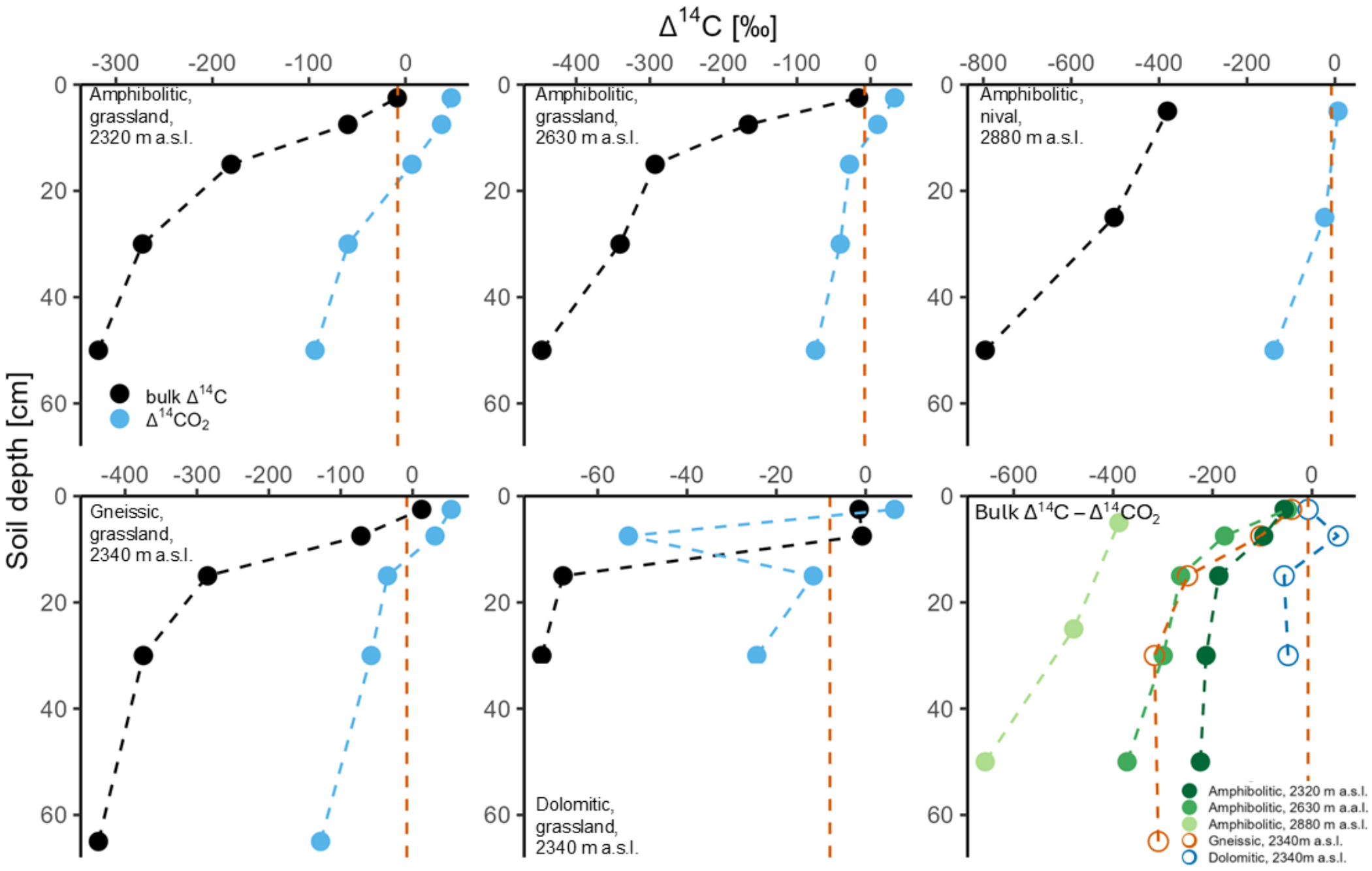

Figure S6: Bulk soil $\Delta^{14}C$ and microbially respired $^{14}CO2$ for selected sites as well as their difference (right bottom panel). Sites were selected to be representative of one small elevation gradient from grassland to nival sites (top panels) as well as to compare bedrock types at similar elevation in grasslands.

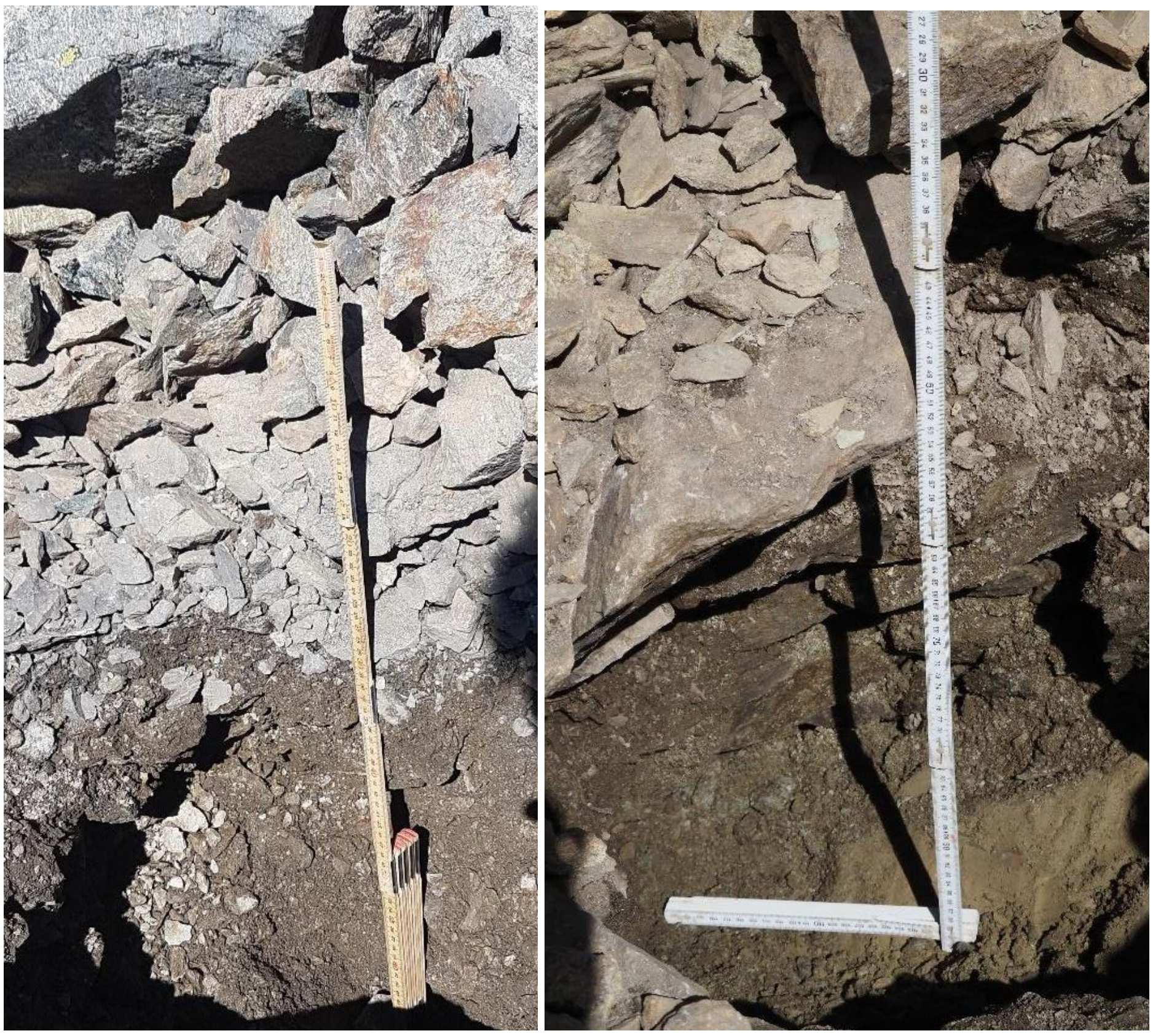

Figure S7: Soil profiles in blockfields of the periglacial region. Left: Schwarzhorn (amphibolitic bedrock); right: Munt Pers (gneissic bedrock)

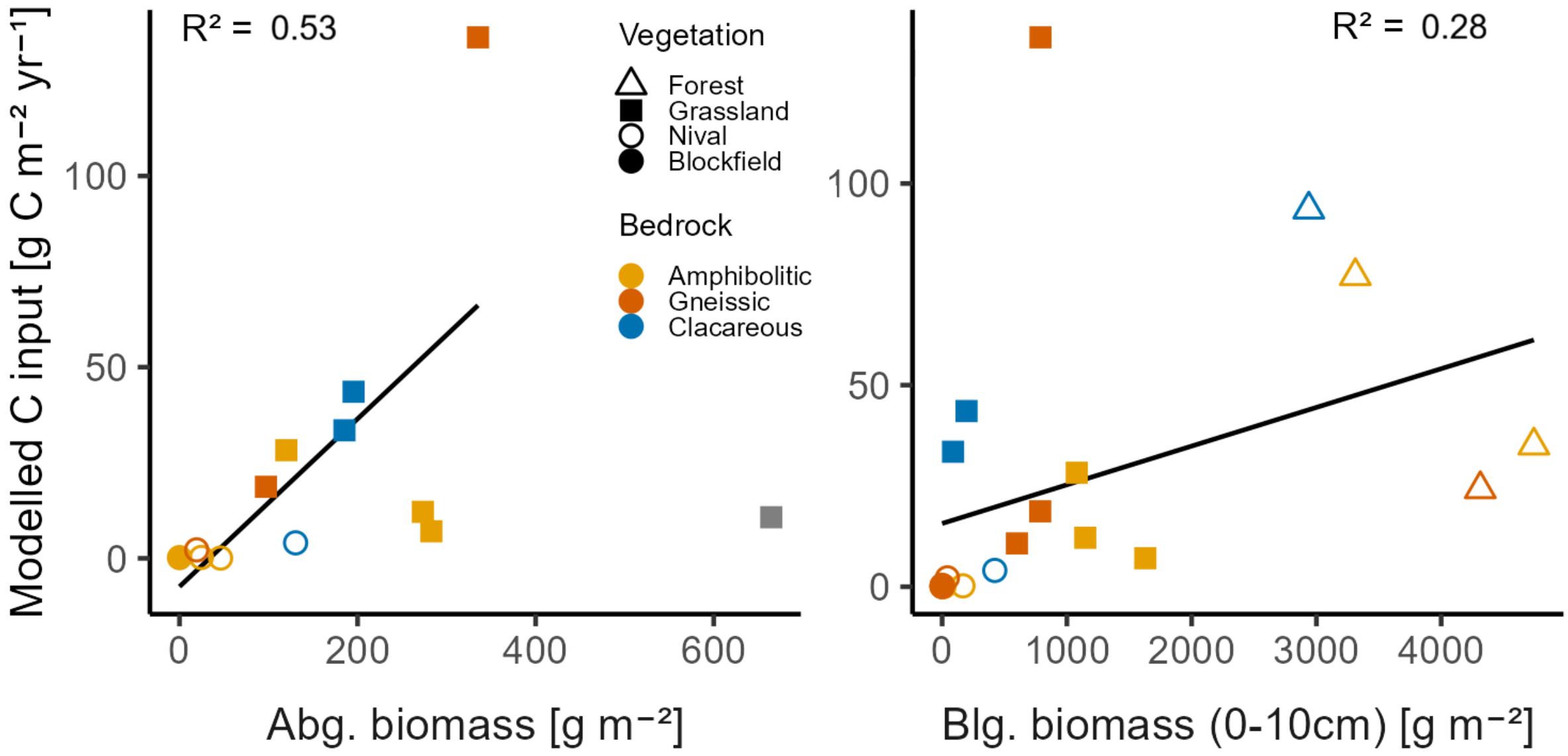

Figure S8: Correlations between aboveground and belowground biomass and modelled C input rates (net C inputs into the soil). For aboveground biomass, forest sites are not shown as only ground biomass was samples, not accounting for tree biomass. Note that an outlier with high aboveground biomass was removed from the linear correlation (shown in grey).

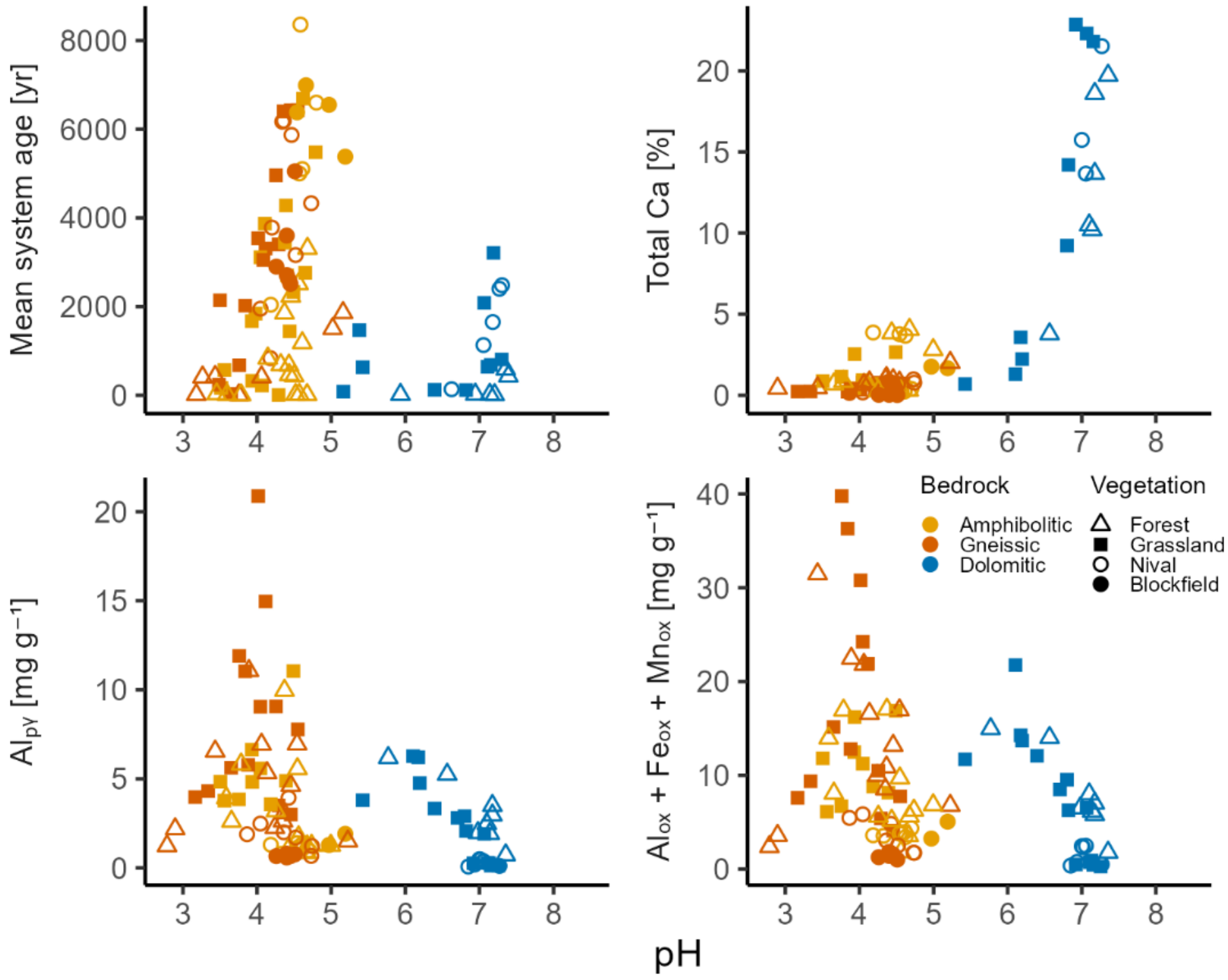

Figure S9: Mean system ages, total Ca and pedogenic oxide concentrations against soil pH

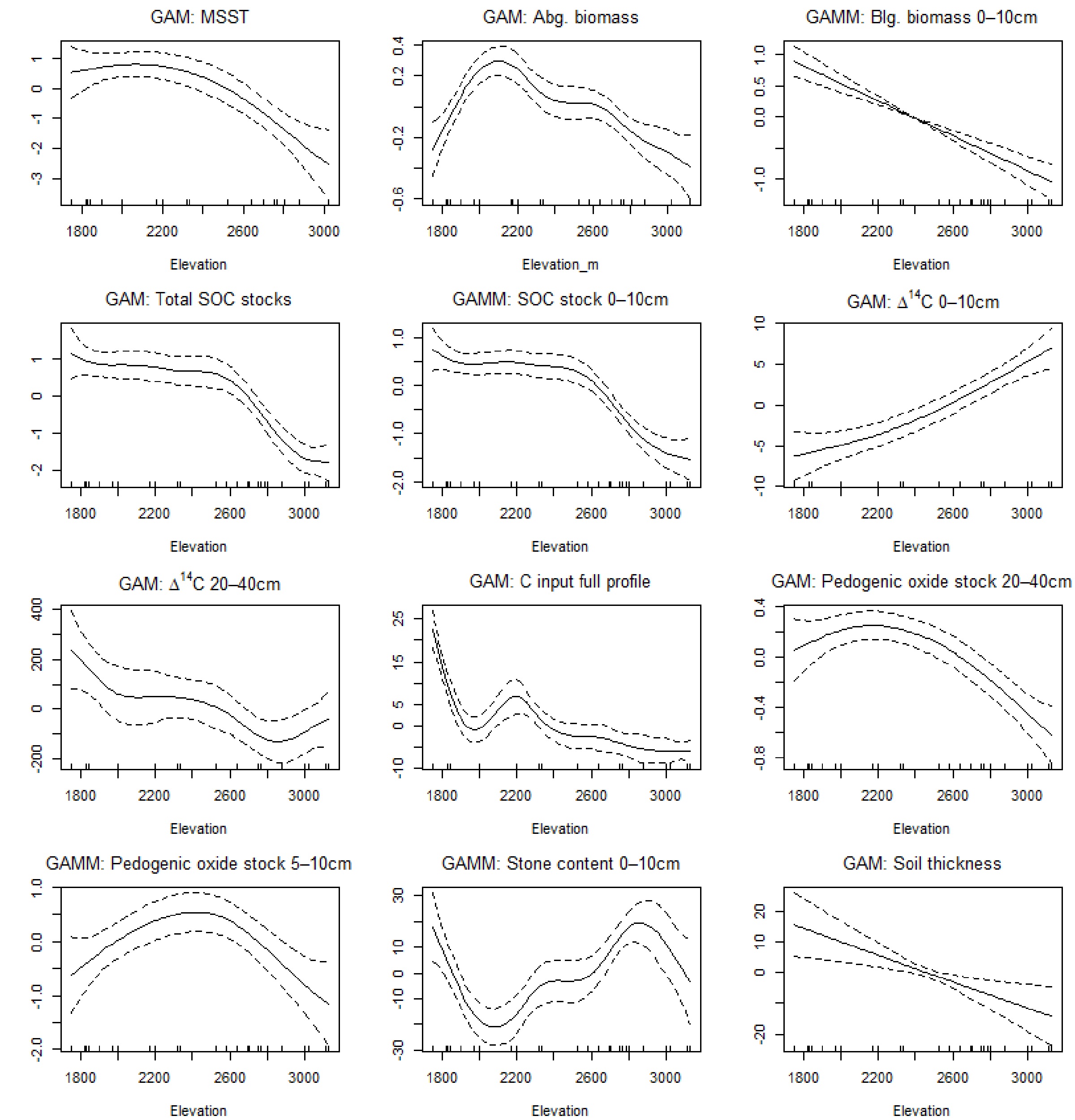

Figure S10: Partial effect plots of gam analysis; edf, F- and p-values can be found in Table S12.

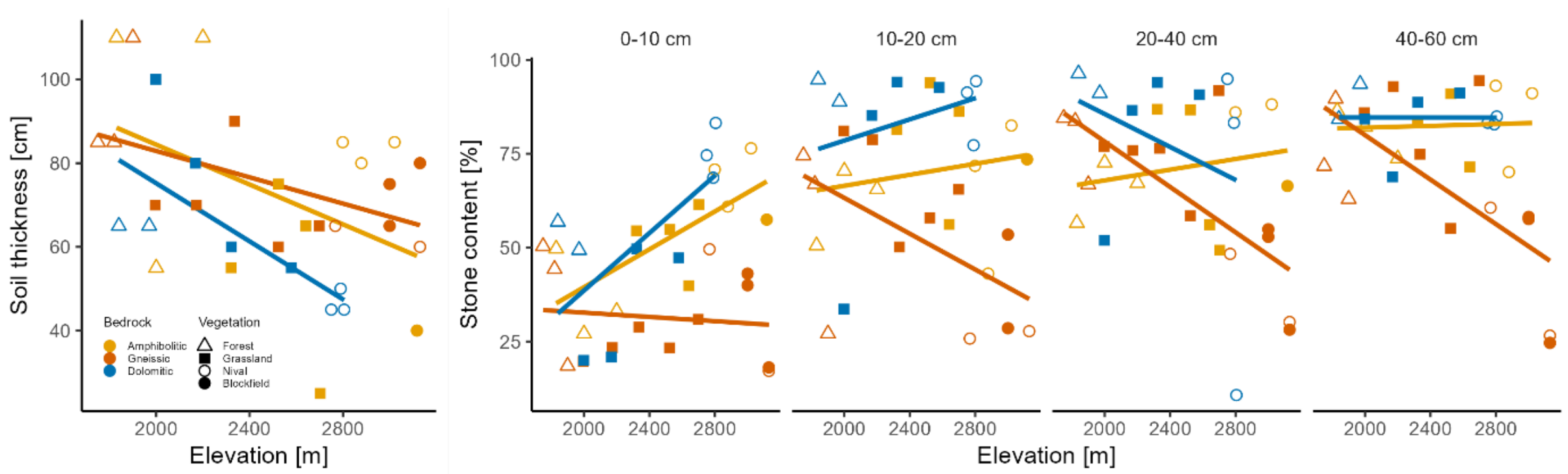

Figure S11: Soil thickness and stone content for different depth intervals along the elevation gradients.

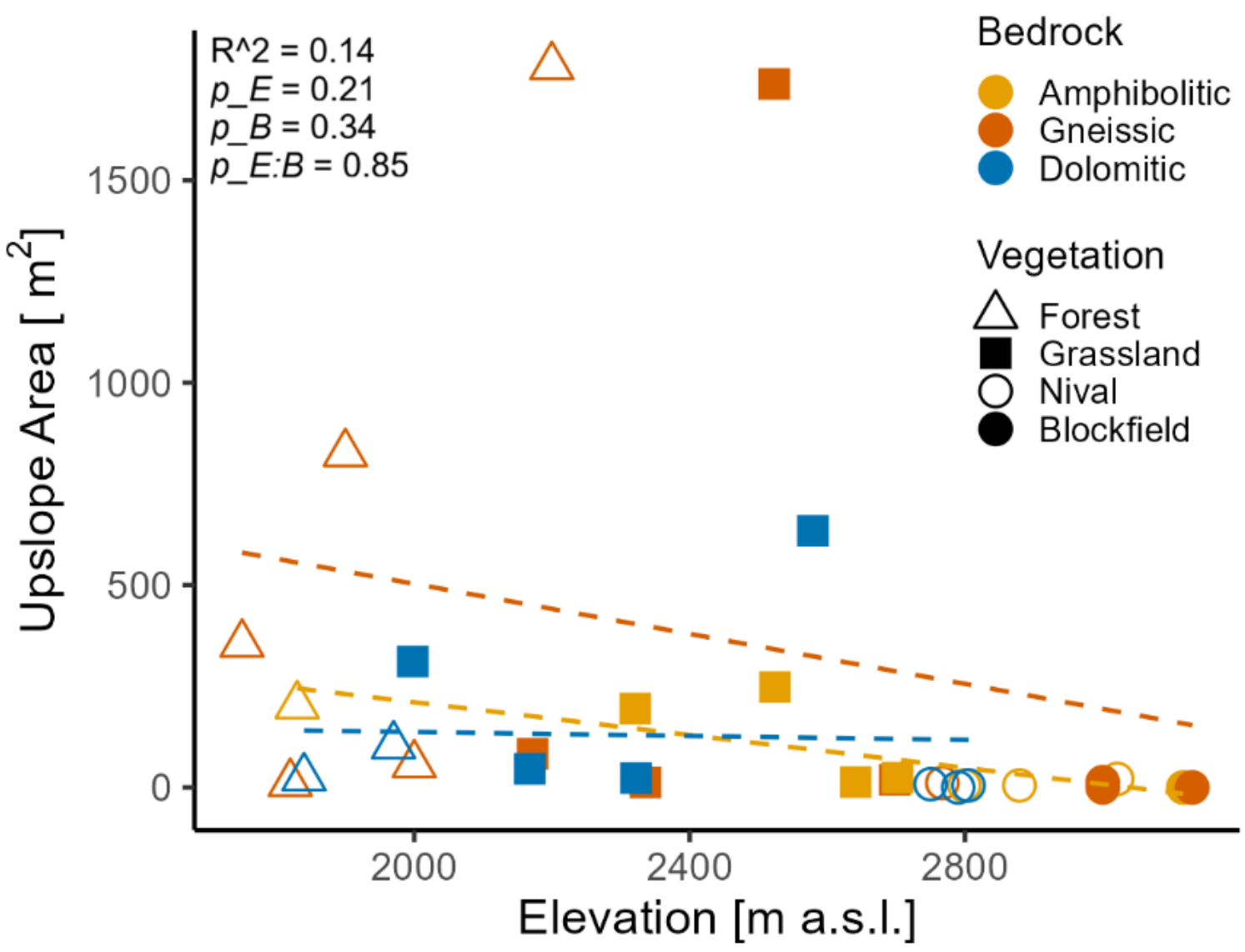

Figure S12: Contributing upslope area plotted against elevation for each bedrock type.

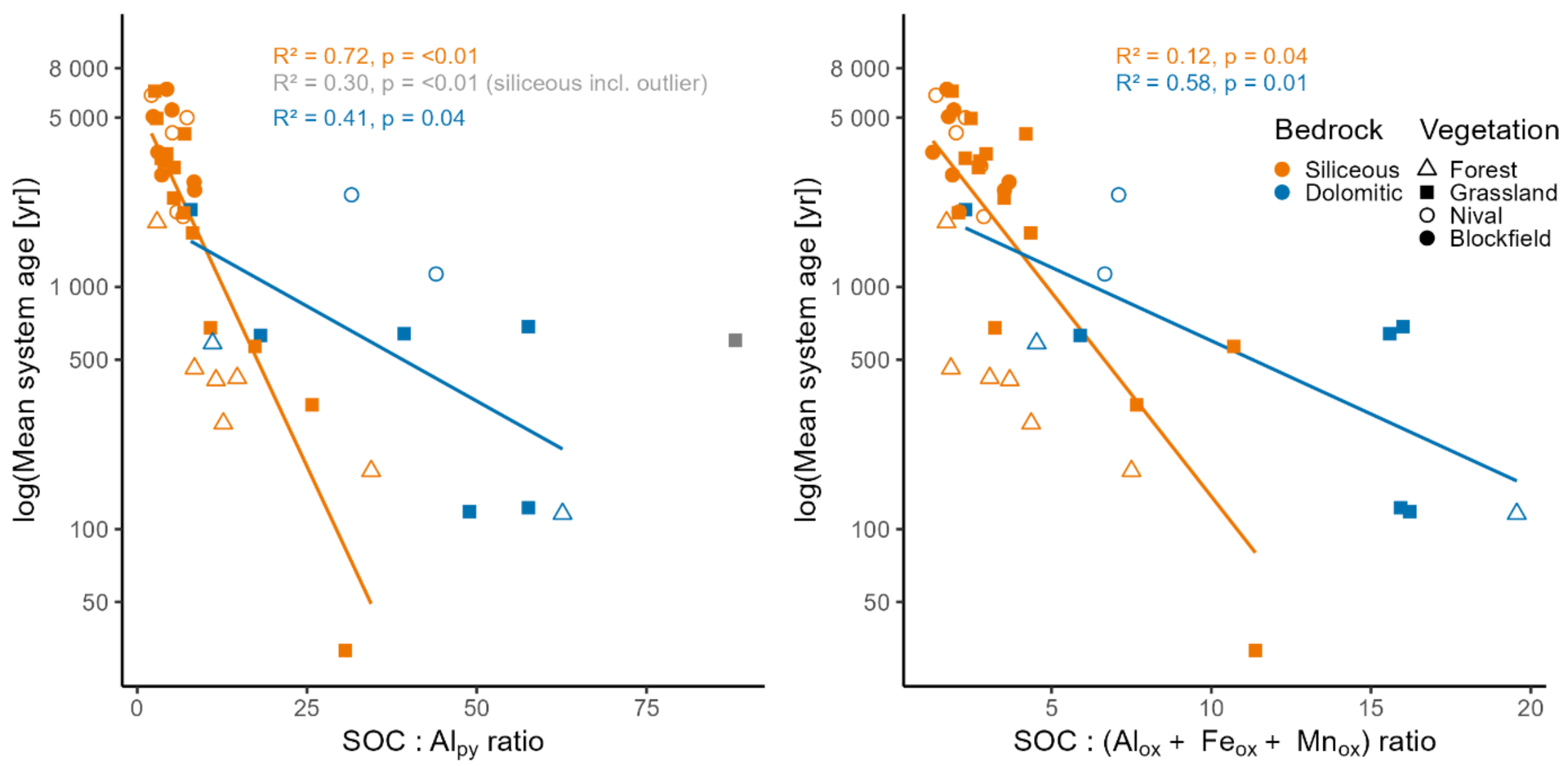

Figure 13: Correlations between mean system age with SOC:Al_{py} and SOC:(Al_{ox} + Fe_{ox} + Mn_{ox}) mass ratios for siliceous and dolomitic bedrock types separately.

Tables

Table S1: Study site overview

Site	Elevation [m asl.]	Slope [°]	Aspect (°N)	Vegetation	Geology[1]	Soil parent material[2]	Sampling date [dd.mm.yyyy]	Sampling depth [cm]	Soil classification
Gradient on gneissic bedrock (Dischma valley – Furka Radönt + Diavolezza)									
DR1750	1750	31.6	266	forest	orthogneiss	till	27.06.2023	85	podsolised Humic Cambisol
DR1820	1820	27.7	236	forest	orthogneiss	till	28.06.2023	85	podsolised Humic Cambisol
DR1900	1900	24.6	216	forest	orthogneiss	till	12.06.2023	110	Humic Cambisol
DR2000	1996	8.1	278	grassland	paragneiss	till	28.06.2022	70	Umbric Regosol
DR2180	2172	22.3	275	grassland	paragneiss	till	30.06.2022	70	Umbric Regosol
DR2340	2336	23.3	196	grassland	paragneiss	till	30.06.2022	90	Umbric Regosol
DR2520	2523	11	184	grassland	composite gneiss	till	19.07.2022	60	Umbric Regosol
DR2690	2698	15.4	165	grassland	composite gneiss	slope waste	16.08.2022	65	Umbric Regosol
DR2780	2767	16.8	117	nival	composite gneiss	solifluction	27.07.2022	65	Ochric Regosol
SQR1	3000	16.7	285	blockfield	granodiorite	blockfield	19.07.2023	75	Regosol
MTP1	3130	16.2	194	blockfield	granodiorite	blockfield	18.07.2023	80	Regosol
MTP2	3130	7.9	63	nival	granodiorite	blockfield	18.07.2023	60	Regosol
Gradient on amphibolitic bedrock (Fluelapass - Schwarzhorn)									
FS1830	1830	28	222	forest	amphibolite	slope waste	26.06.2023	110	podsolised Humic Cambisol
FS2000	2000	24.7	101	forest	orthogneiss	slope waste	26.06.2023	55	Humic Cambisol
FS2200	2200	28.8	201	forest	amphibolite	till	13.06.2023	110	podsolised Humic Cambisol
FS2320	2321	14	46	grassland	orthogneiss	till	28.07.2022	55	Umbric Regosol
FS2520	2524	36.5	106	grassland	amphibolite	slope waste	26.07.2022	75	Umbric Regosol
FS2630	2641	27.5	137	grassland	amphibolite	slope waste/till	27.07.2022	65	Umbric Regosol
FS2690	2702	35.1	57	grassland	amphibolite	slope waste/till	27.07.2022	25	Umbric Regosol
FS2800	2799	25.8	335	nival	amphibolite	till	07.07.2023	85	Ochric Regosol
FS2880	2879	16.7	201	nival	amphibolite	blockfield	13.07.2022	80	Ochric Regosol
FS3040	3021	19.5	286	nival	amphibolite	blockfield	13.07.2022	85	Ochric Regosol
FS3100	3117	14.4	115	blockfield	amphibolite	blockfield	13.07.2022	40	Regosol
Gradient on dolomitic bedrock (Inneralp – Büelenhorn)									
IB1840	1840	27.2	256	forest	main dolomite	till	27.06.2023	65	Calcaric Cambisol

IB1970	1970	27.4	192	forest	main dolomite	till	27.06.2023	65	Calcaric Cambisol
IB2000	1998	12.6	15	grassland	main dolomite	slope waste/till	27.06.2022	100	Calcaric Cambisol
IB2180	2168	13.7	50	grassland	main dolomite	till	27.06.2022	80	Regosol
IB2340	2322	21.3	31	grassland	main dolomite	till	07.07.2022	60	Regosol
IB2570	2579	26.9	83	grassland	main dolomite	slope waste	08.07.2022	55	Regosol
IB2750	2750	14.3	72	nival	main dolomite	breccia	08.07.2022	45	Regosol
IB2790	2790	26.9	331	nival	main dolomite	breccia	22.08.2024	50	Regosol
IB2800	2805	31.7	30	nival	main dolomite	breccia	22.08.2024	45	Regosol

1 – SwissTopo: Geologische Karte der Schweiz 1:25 000, 2005

2 – SwissTopo: GeoCover, 2023

Table S2: Air temperature references for MAAT estimation along the elevation gradients (IMIS, 2023). MAAT for each sampling site was calculated using an adiabatic temperature gradient of 0.5°C per 100 m in elevation.

IMIS station ID	Start year data recording	Elevation [m a.s.l.]	MAAT [°C]	Reference for gradient
FLU2	2004	2394	0.4	amphibolitic & gneissic
BER3	2001	2625	0.0	gneissic > 3000 m
DAV2	1999	2558	-0.1	dolomitic

Table S3: Dominant plant species for grassland and nival sites

Vegetation type	Amphibolitic		Gneissic		Dolomitic	
	Species	Cover	Species	Cover	Species	Cover
Grassland	*Lotus alpinus*	35	*Erica carnea*	30	*Nardus stricta*	52
	Salix herbacea	33	*Dryas octopetala*	28	*Loiseleuria procumbens*	22
	Carex curvula	14	*Carex sempervirens*	19	*Rhododendron ferrugineum*	16
	Carex sempervirens	13	*Sesleria caerulea*	19	*Festuca halleri aggr.*	15
	Geum montanum	5	*Polygala chamaebuxus*	12	*Juniperus communis subsp. alpina*	15
	Soldanella alpina	5			*Vaccinium gaultherioides*	15
Nival	*Minuartia sedoides*	6	*Sesleria caerulea*	8	*Gnaphalium supinum*	5
	Poa alpina	3	*Agrostis alpina*	7	*Lichens*	2
	Ranunculus glacialis	3	*Silene acaulis*	6	*Poa alpina*	2
	Carex spicata	2	*Arenaria ciliata*	1	*Leucanthemopsis alpina*	1
	Saxifraga bryoides	1	*Saxifraga exarata subsp. moschata*	1	*Euphrasia minima*	1

Table S4: Model parameters and model evaluation for linear and sigmoidal models representing total SOC stocks with elevation. The parameters of the sigmoidal model refer to equation (6).

Bedrock	Model	Model parameter					AIC	R^2*
		Slope	Intercept	Asym	Xmid	Scal		
Amphibolitic	Linear	-0.0016	6.3				30.2	0.38
	Sigmoidal			9.1	2876.1	-156.4	27.5	0.64
Gneissic	Linear	-0.0024	8.6				32.0	0.75
	Sigmoidal			14.8	2857.6	-144.9	23.1	0.90
Dolomitic	Linear	-0.0018	6.4				1.9	0.91
	Sigmoidal			8.7	2630.7	-289.31	0.6	0.95

* pseudo-R^2 for sigmoidal model

Table S5: Lag times which were applied to buffer the atmospheric ^{14}C curve prior to mean system age calculation. Constrained after Leifeld et al., 2015 (alpine ecosystems) and Solly et al., 2013 (forest ecosystems) as well as logical implications from the bulk soil ^{14}C content.

Vegetation type	Elevation [m a.s.l.]	Depth [cm]	Lag time [yr]
forest		organic layer	0.1
forest		0 – 10	1.4
forest		10 – 20	6.7
forest		> 20	10.7
grassland	< 2500	0 – 10	7
grassland	< 2500	> 10	8.5
grassland	> 2500	0 – 10	9
grassland	> 2500	> 10	11.5
nival		0 – 10	12
nival		> 10	14.5
none		0 – 60	0

Table S6: Laboratory incubation for ^{14}C analysis of the microbially respired CO_2

Site	Depth [cm]	pH	TOC [%]	TC [%]	$\delta^{13}C$ [‰]	$\Delta^{14}C$ [‰]	Respiration rate [mgC/h]	$\Delta^{13}CO_2$ [‰]	$\Delta^{14}CO_2$ [‰]
IB2340	2.5	6.4	19.23	24.97	-24.7	-1.46	67.9	-26.5	6.50
IB2340	7.5	6.8	10.15	17.86	-24.6	-0.76	34.4	-24.1	-53.14
IB2340	15	7.1	1.13	13.51	-25.6	-67.77	28.2	-25.7	-11.72
IB2340	30	7.2	0.72	12.99	-25.6	-72.53	12.7	-23.3	-24.38
FS2630	2.5	4.1	9.84	9.84	-26.1	-16.32	32.6	-26.5	32.75
FS2630	7.5	3.9	5.42	5.42	-25.3	-166.10	12.2	-25.7	9.72
FS2630	15	4.4	3.28	3.28	-25.4	-292.77	9.3	-24.6	-28.55
FS2630	30	4.4	3.42	3.42	-25.1	-340.45	9.0	-24.0	-41.23
FS2630	50	4.6	0.47	0.47	-25.3	-446.81	1.7	-18.6	-74.85
DR2340	2.5	3.7	17.24	17.24	-25.9	12.62	65.3	-26.3	53.78
DR2340	7.5	3.8	12.84	12.84	-25.9	-71.83	21.9	-25.6	31.22
DR2340	15	4.1	6.03	6.03	-25.2	-285.24	5.7	-23.3	-34.98
DR2340	30	4.3	2.6	2.6	-25.6	-374.55	6.7	-23.0	-57.64
DR2340	65	4.5	0.77	0.77	-25.8	-437.09	0.6	-22.5	-128.00
FS2320	2.5	3.5	10.35	10.35	-26.0	-8.49	76.9	-26.1	47.50
FS2320	7.5	3.6	6.54	6.54	-25.6	-59.64	20.8	-25.2	37.47
FS2320	15	4.0	3.97	3.97	-26.2	-180.67	15.7	-24.9	6.82
FS2320	30	4.0	3.05	3.05	-26.0	-272.45	6.8	-25.7	-59.38
FS2320	50	4.1	3.27	3.27	-25.3	-318.15	15.5	-23.4	-93.69
FS2880	5	4.6	1.54	1.54	-26.3	-381.19	9.5	-24.9	7.03
FS2880	25	4.6	0.62	0.62	-26.4	-502.41	2.6	-24.0	-23.26
FS2880	60	4.7	0.32	0.32	-28.6	-795.71	0.7	-	-138.46

Table S7: Measured soil respiration (SR) in the field. Annual fluxes were estimated after Bahn et al, 2010. Annual T soil from TMS loggers during 2022-2024, when available.

Site	September 2022		July 2023		August 2023		September 2023		Annual	
	CO_2 flux [mgCO_2-C/m^2/h]	T soil [°C]	CO_2 flux [mgCO_2-C/m^2/h]	T soil [°C]	CO_2 flux [mgCO_2-C/m^2/h]	T soil [°C]	CO_2 flux [mgCO_2-C/m^2/h]	T soil [°C]	CO_2 flux [mgCO_2-C/m^2/h]	T soil [°C]
Gneissic										
DR1750	-	-	196.4 ± 43.7	15.0	351.9 ± 108.6	9.8	213.8 ± 45	11.2	1092.9	5.5
DR1820	-	-	202.1 ± 38.9	14.6	195.5 ± 48.4	10.1	160.7 ± 12.4	10.6	806.0	5.3
DR1900	-	-	262.9 ± 26	15.0	265.8 ± 89.3	10.8	256.9 ± 71.7	12.9	1009.5	5.4
DR2000	135.9 ± 66.9	-	249.4 ± 24.4	18.3	189 ± 46	8.1	139.8 ± 22.7	14.3	669.0	5.8
DR2180	96.1 ± 17.3	-	308.8 ± 55.4	17.6	214.4 ± 47.9	10.6	141.3 ± 15.2	12.4	656.2	5.1
DR2340	46.1 ± 78.2	-	218.8 ± 47.3	17.0	231.7 ± 54.7	11.0	146.5 ± 54	12.2	565.8	5.1
DR2520	69.7 ± 16.7	-	149.2 ± 30.0	13.7	130.8 ± 6.5	8.3	114.9 ± 26.4	11.5	-	-
DR2690	40.8 ± 4.1	-	61.4 ± 9.8	10.1	56.4 ± 15.1	6.1	104 ± 12.3	10.0	321.1	3.4
DR2780	95.6 ± 21.8	-	49.5 ± 25.7	16.3	14.9 ± 3.7	2.5	31.2 ± 12	5	240.9	2.9
Amphibolitic										
FS1830	-	-	159.2 ± 24.6	14.3	106.1 ± 12	8.5	280.5 ± 73.7	11.6	836.6	5.6
FS2000	-	-	-	-	71.5 ± 9.6	10.7	169.5 ± 39.9	13.2	-	-
FS2200	-	-	165.8 ± 41	18.5	42.8 ± 9.1	5.5	177.5 ± 20.8	16.8	445.9	5.1
FS2320	109.1 ± 6.9	14.7	101.6 ± 14.5	21.2	15.9 ± 5.2	3.9	132.6 ± 23.3	12.1	271.8	3.3
FS2520	145.3 ± 1.8	17.1	274.1 ± 74.2	18.2	138.5 ± 15.6	7.6	226 ± 18.3	17.4	-	-
FS2630	110 ± 5.4	16.2	268.7 ± 25.2	20.7	96 ± 15.8	6.2	263.1 ± 41	13.4	565.9	4.6
FS2690	-	-	108.7 ± 25.5	19.4	73.5 ± 15.4	5.2	59.9 ± 7	10.6	-	-
FS2800	-	-	15.6 ± 4.2	27.1	10.2 ± 5.6	5.2	6.7 ± 5.1	14.5	-	-
FS2880	-	-	5.8 ± 2.1	20.5	7 ± 3.2	3.4	8.4 ± 3.1	3.1	12.8	-0.3
FS3040	7.2 ± 2.3	14.6	5.2 ± 6.5	26.4	0.8 ± 3	0.5	8.7 ± 1	14.7	12.4	1.8
FS3100	3.4 ± 0.3	-	28.7	11.6	8.9 ± 7.4	0.8	-6.2 ± 7.6	4.7	45.2	1.0
Dolomitic										
IB1840	-	-	212.3 ± 29.6	14.8	200.8 ± 24.4	9.7	370.6 ± 161.8	10.6	1176.9	5.6
IB1970	-	-	277.8 ± 119.8	15.0	306.6 ± 139.6	11.0	171.9 ± 30.2	10.7	-	-
IB2000	171.7 ± 37.1	14.5	338.5 ± 51.1	19.4	269.2 ± 46.3	14.5	266.6 ± 49.4	12.3	769.0	5.2
IB2180	163.6 ± 55.5	14.5	347 ± 72.7	18.3	263.9 ± 46.2	13.2	126.1 ± 13.5	11.3	667.7	4.5
IB2340	76.8 ± 11.7	15.5	173.2 ± 45.8	17.6	202.4 ± 13.7	10.7	188.1 ± 57.7	10.1	511.3	4.3
IB2570	141 ± 5.1	15.3	266 ± 77.3	20.1	171.6 ± 28.1	7.8	89.7 ± 56.6	10.9	-	-
IB2750	58.3 ± 19.6	14.6	72.5 ± 12.3	21.9	47.3 ± 7.1	5.3	96.2 ± 5.6	8.2	215.0	3.1
IB2800	21.4 ± 2.7	11.5	47.5	21.0	19.9 ± 8.2	2.1	27.2 ± 4.7	8.6	97.3	2.0

Table S8: TC and TN contents in selected samples on amphibolitic and gneissic bedrock

Site	Bedrock	Depth [cm]	TC [%]	TN [%]	C:N
FS3100	amphibolitic	0-20	0.010	0.034	3.4
FS3100	amphibolitic	40-70	0.010	0.040	4.0
MTP1	gneissic	45-55	0.008	0.057	7.1
MTP1	gneissic	105-120	0.005	0.025	5.0
SQR1	gneissic	0-5	0.009	0.049	5.4
SQR1	gneissic	20-40	0.007	0.036	5.1

Table S9: Statistical results of linear models and linear mixed-effect models for the presented data. Both models use elevation, bedrock and their interaction as explanatory variables. When more than one field replicate was available, the statistical model was fixed for site effects. Effect sizes are reported using semi-partial R^2 (sr2) for linear models and η^2 for mixed effect models. Abbr.: Abg. – aboveground; Blg. – belowground; MSST – mean summer soil temperature for months July, August and September (for available years 2022-2024); Cmin. – C mineralization upon lab incubation, FE – fine earth.

Topography

	Slope[1]			Exposure[1]			Upslope Area[1]		
	sr2	*F*	*p*	*sr2*	*F*	*P*	*sr2*	*F*	*p*
Elevation	0.03	3.806	0.062	0.01	0.384	0.541	<0.01	1.627	0.214
Bedrock	0.09	3.249	0.055	0.07	2.500	0.101	0.02	1.126	0.340
Elevation: Bedrock	0.12	1.919	0.167	0.05	1.007	0.379	0.01	0.159	0.854

Vegetation

	Abg. biomass[1]			Blg. Biomass 0-10cm[2]			MSST[1]		
	sr2	*F*	*p*	*η2*	*F*	*p*	*sr2*	*F*	*p*
Elevation	0.02	6.419	**0.019**	0.61	46.225	**<0.001**	0.17	16.583	**<0.001**
Bedrock	0.02	0.020	0.980	0.05	0.737	0.487	0.06	0.186	0.831
Elevation: Bedrock	0.02	0.520	0.602	0.03	0.370	0.694	0.06	0.513	0.606

Soil characteristics

	pH 0-10cm[1]			Soil thickness[1]			FE mass (0-10cm)[2]			Stone content (0-10cm)[2]		
	sr2	*F*	*p*	*sr2*	*F*	*p*	*η2*	*F*	*p*	*η2*	*F*	*p*
Elevation		0.010	0.921	0.08	7.223	**0.012**	0.19	5.371	**0.030**	0.25	9.287	**0.005**
Bedrock		63.801	**<0.001**	0.01	1.960	0.160	0.02	0.250	0.781	0.16	2.479	0.103
Elevation: Bedrock		0.193	0.926	0.02	0.480	0.624	0.03	0.310	0.736	0.23	4.203	**0.025**

	Stone content (10-20cm)[1]			Stone content (20-40cm)[1]			Stone content (40-60cm)[1]		
	sr2	*F*	*p*	*sr2*	*F*	*p*	*sr2*	*F*	*p*
Elevation	<0.01	0.595	0.446	<0.01	5.205	**0.030**	0.01	17.202	**<0.001**
Bedrock	0.07	5.920	**0.007**	0.10	6.045	**0.006**	0.18	2.328	0.128
Elevation: Bedrock	0.13	3.125	0.058	0.16	4.500	**0.019**	0.23	5.781	**0.012**

	Pedogenic oxide stock 5-10cm[2]			Pedogenic oxide stock 20-40cm[1]			SOC:PO 5-10cm[2]			Clay content (5-10cm)[1]		
	η2	*F*	*p*	*sr2*	*F*	*p*	*η2*	*F*	*p*	*sr2*	*F*	*p*
Elevation	0.04	0.953	0.338	0.12	40.104	**<0.001**	0.47	4.828	**0.037**	0.12	40.104	**<0.001**
Bedrock	0.02	0.213	0.809	0.05	3.640	**0.040**	0.06	1.721	0.199	0.05	3.640	**0.040**
Elevation: Bedrock	0.03	0.347	0.710	0.03	1.291	0.292	0.09	1.348	0.277	0.03	1.291	0.292

SOC stocks & radiocarbon content

	Total SOC stock[1]			Mineral SOC stock[1]			SOC stock 0-10 cm[2]		
	sr2	*F*	*p*	*sr2*	*F*	*P*	*η2*	*F*	*p*
Elevation	0.11	56.175	**<0.001**	0.1	42.013	**<0.001**	0.66	56.96	**<0.001**
Bedrock	0.03	5.955	**0.007**	0.03	4.970	**0.015**	0.02	0.216	0.807
Elevation: Bedrock	0.02	0.800	0.430	0.02	0.660	0.525	0.02	0.308	0.737

	$\Delta^{14}C$ 0-10cm[1]			$\Delta^{14}C$ 20-40cm[1]			$\Delta^{14}C$ 60-80cm[1]		
	sr2	*F*	*p*	*sr2*	*F*	*p*	*sr2*	*F*	*p*
Elevation	0.47	49.927	**<0.001**	0.24	47.723	**<0.001**	0.61	12.628	**0.006**
Bedrock	0.09	0.988	0.397	0.03	7.675	**0.002**	0.17	2.322	0.154
Elevation: Bedrock	0.08	2.989	0.083	0.04	3.529	**0.041**	0.15	3.503	0.075

C input & field CO_2 fluxes

	Modelled C input (full profile)[1]			CO_2 flux[2]			R10[2]			R10:SOC[2]		
	sr2	*F*	*p*	*η2*	*F*	*p*	*η2*	*F*	*P*	*η2*	*F*	*p*
Elevation	0.19	9.400	**0.008**	0.66	43.067	**<0.001**	1.00	541.384	0.220	0.94	3.604	0.088
Bedrock	0.03	1.493	0.258	0.02	0.284	0.755	0.91	10.112	0.090	0.99	18.708	**0.004**
Elevation: Bedrock	0.02	0.370	0.370	0.01	0.127	0.881	0.94	14.401	0.065	0.99	51.561	**<0.001**

SOC quality

	C:N 0-10cm[2]			C:N 0-10cm above treeline[2]			C min. per g dry mass[2]			C min. per g SOC[2]		
	η2	*F*	*p*	*η2*	*F*	*P*	*η2*	*F*	*p*	*η2*	*F*	*p*
Elevation	0.47	24.407	**<0.001**	0.40	13.897	**0.001**	0.20	5.904	**0.023**	0.02	0.364	0.552
Bedrock	<0.01	0.106	0.900	0.09	1.030	0.375	0.01	0.129	0.879	0.31	5.208	**0.014**
Elevation: Bedrock	0.02	0.305	0.739	0.03	0.272	0.765	0.03	0.357	0.704	0.39	7.250	**0.004**

1 – linear model (n = 1)

2 – linear mixed effect model (n > 1)

bold – p < 0.05, interpreted as statistically significant

Table S10: Statistical analysis for a linear model explaining SOC stocks with elevation, bedrock and depth. SOC depths were square root transformed to achieve normal distribution.

	F	*p*
Elevation	179.682	**<0.001**
Bedrock	28.896	**<0.001**
Depth	11.8441	**<0.001**
Elevation:Bedrock	1.234	0.293
Elevation:Depth	0.732	0.645
Bedrock:Depth	1.470	0.127
Elevation:Bedrock:Depth	3.113	**<0.001**

Table S11: Statistical analysis for a linear model explaining modelled C inputs with elevation, bedrock and depth. C input rates was log transformed to achieve normal distribution.

	F	*p*
Elevation	194.849	**<0.001**
Bedrock	2.121	0.129
Depth	17.848	**<0.001**
Elevation:Bedrock	0.866	0.426
Elevation:Depth	4.351	**<0.001**
Bedrock:Depth	1.123	0.358
Elevation:Bedrock:Depth	1.335	0.240

Table S12: Statistical results of generalized additive models (GAM) and generalized additive mixed model (GAMM). Both models use elevation and bedrock as explanatory variables where elevation was fitted using the smooth function s(). When more than one field replicate was available, the gam was fixed for site effects. We only report F- and p-values as effect sizes are misleading for non-linear models. Abbr.: MSST – mean summer soil temperature for months July, August and September (for available years 2022-2024); Abg. – aboveground; Blg. – belowground.

Model	Term	Term type	edf	*F*	*p*
MSST[1]	Elevation	smooth	2.26	9.87	< 0.001
	Bedrock	parametric	-	0.11	0.900
Abg. biomass[1]	Elevation	smooth	5.57	9.87	< 0.001
	Transect	parametric	-	0.72	0.498
Blg. biomass 0-10cm[2]	Elevation	smooth	1	52.76	< 0.001
	Bedrock	parametric	-	3.01	0.055
Total SOC stocks[1]	Elevation	smooth	5.16	21	< 0.001
	Bedrock	parametric	-	13.32	< 0.001
SOC stock 0-10cm[2]	Elevation	smooth	4.76	28.39	< 0.001
	Bedrock	parametric	-	6.44	0.003
$\Delta^{14}C$ 0-10cm[1]	Elevation	smooth	1.72	17.99	< 0.001
	Bedrock	parametric	-	0.66	0.529
$\Delta^{14}C$ 20-40cm[1]	Elevation	smooth	4.09	3.65	0.02
	Bedrock	parametric	-	3.36	0.059
C input full profile[1]	Elevation	smooth	7.72	21.12	< 0.001
	Bedrock	parametric	-	0.46	0.646
Pedogenic oxide stock 5-10cm[2]	Elevation	smooth	2.85	5.13	0.003
	Bedrock	parametric	-	2.15	0.123
Pedogenic oxide stock 20-40cm[2]	Elevation	smooth	2.49	11.44	< 0.001
	Bedrock	parametric	-	33.59	< 0.001
Stone content 0-10cm[2]	Elevation	smooth	5.75	10.96	< 0.001
	Bedrock	parametric	-	14.64	< 0.001
Soil thickness[1]	Elevation	smooth	1.03	8.57	0.006
	Bedrock	parametric	-	2.02	0.151

1 – GAM; 2- GAMM

Table S13: Statistical testing of contributing upslope area on SOC stocks and radiocarbon contents. Upslope area was calculated using a 2m DEM (swissALTI3D, swisstopo 2019). The basic model (as reported in Table S9) was compared against an extended model including the upslope area (uparea) using a nested model comparison (F-test) and report size effect, F- and p-value for the contributing upslope area in the extended model.

Variable	Model	Fixed effects	AIC	R^2 [1]	Model comparison		Contributing upslope area		
					Test stat.[2]	*p*	Size effect[3]	F	*p*
Total SOC stock[1]	Linear	e * b	76.30	0.70	-	-	-	-	-
		e * b + uparea	77.17	0.71	0.88	0.36	0.01	1.05	0.32
Mineral SOC stock[1]	Linear	e * b	78.29	0.64	-	-	-	-	-
		e * b + uparea	78.55	0.66	1.40	0.25	0.02	1.58	0.22
SOC stock 0-10 cm[2]	Linear mixed effect	e * b	169.81	0.85	-	-	-	-	-
		e * b + uparea	195.56	0.85	1.24	0.26	0.04	0.99	0.33
$\Delta^{14}C$ 0-10 cm[1]	Linear	e * b	113.73	0.72	-	-	-	-	-
		e * b + uparea	115.50	0.72	0.15	0.70	<0.01	0.21	0.66
$\Delta^{14}C$ 10-20 cm[1]	Linear	e * b	192.94	0.81	-	-	-	-	-
		e * b + uparea	191.85	0.84	1.91	0.20	0.03	1.75	0.22
$\Delta^{14}C$ 20-40 cm[1]	Linear	e * b	307.62	0.53	-	-	-	-	-
		e * b + uparea	308.78	0.55	0.60	0.45	0.02	0.84	0.37
$\Delta^{14}C$ 60-80 cm[1]	Linear	e * b	161.10	0.75	-	-	-	-	-
		e * b + uparea	157.38	0.84	3.05	0.14	0.10	4.08	0.08

1 – Pseudo-R^2 for linear mixed effect models

2 – test statistic: F-values for linear models, X^2 for linear mixed effect models

3 – size effects: sr^2 for linear modes, η^2 for linear mixed effect models

Table S14: Statistical output for the categorical model testing whether SOC stocks differ by vegetation line and bedrock. Sites were assigned to above- or below-vegetation-line groups using the threshold derived from the sigmoidal model (Table S2), and the model was specified as lm(SOC ~ veg.line × bedrock).

	Total SOC stocks			Mineral SOC stocks		
	sr2	*F*	*p*	*sr2*	*F*	*p*
Veg.line	0.09	79.271	**<0.001**	0.09	75.260	**<0.001**
Bedrock	<0.01	3.806	**0.035**	<0.01	3.985	**0.031**
Veg.line : Bedrock	0.07	3.691	**0.038**	0.07	3.993	**0.030**

References

Bahn, M., Reichstein, M., Davidson, E. A., Grünzweig, J., Jung, M., Carbone, M. S., Epron, D., Misson, L., Nouvellon, Y., Roupsard, O., Savage, K., Trumbore, S. E., Gimeno, C., Curiel Yuste, J., Tang, J., Vargas, R., & Janssens, I. A. (2010). Soil respiration at mean annual temperature predicts annual total across vegetation types and biomes. *Biogeosciences*, *7*(7), 2147–2157. https://doi.org/10.5194/bg-7-2147-2010

Leifeld, J., Meyer, S., Budge, K., Sebastia, M. T., Zimmermann, M., & Fuhrer, J. (2015). Turnover of Grassland Roots in Mountain Ecosystems Revealed by Their Radiocarbon Signature: Role of Temperature and Management. *PLOS ONE*, *10*(3), e0119184. https://doi.org/10.1371/journal.pone.0119184

Solly, E., Schöning, I., Boch, S., Müller, J., Socher, S. A., Trumbore, S. E., & Schrumpf, M. (2013). Mean age of carbon in fine roots from temperate forests and grasslands with different management. *Biogeosciences*, *10*(7), 4833–4843. https://doi.org/10.5194/bg-10-4833-2013

SwissTopo Bundesamt für Landestopografie Schweiz. (2023). *Geological Map of Switzerland 1:500.000 -* GeoKarten 500 Vektor [Data set]. SwissTopo. https://www.swisstopo.admin.ch/de/geokarten-500-vektor

SwissTopo Bundesamt für Landestopografie Schweiz. (2024). *Geological Map of Switzerland 1:25.000 Pixel* [Data set]. SwissTopo. https://www.swisstopo.admin.ch/de/geologischer-atlas-der-schweiz-1-25000-pixel

SwissTopo Bundesamt für Landestopografie Schweiz, Federal Office of Topography Switzerland. (2019). *swissALTI3D* [Dataset]. SwissTopo. https://www.swisstopo.admin.ch/de/hoehenmodell-swissalti3d
